# Supplementary material for: Impaired Complex I dysregulates neural/glial precursors and corpus callosum development revealing postnatal defects in Leigh syndrome mice
Source: EMBO Mol Med. 2025 Dec 22;18(2):677–701. doi: 10.1038/s44321-025-00367-4 (PMC12905379; doi:10.1038/s44321-025-00367-4)
Supplement: Supplementary file 1 — Appendix [file 44321_2025_367_MOESM1_ESM.pdf]

Impaired Complex I dysregulates neural/glial precursors and corpus callosum development revealing postnatal defects in Leigh Syndrome mice

Sahitya Ranjan Biswas, Porter L. Tomsick, Colin Kelly, Brooke A. Lester, Julia P. Milner, Sara N. Henry, Yaris Soto, Samantha Brindley, Nicole DeFoor, Paul D. Morton*, and Alicia M. Pickrell*

APPENDIX

Table of Contents

1. Appendix Figure S1 Page 2
2. Appendix Figure S2 Page 3

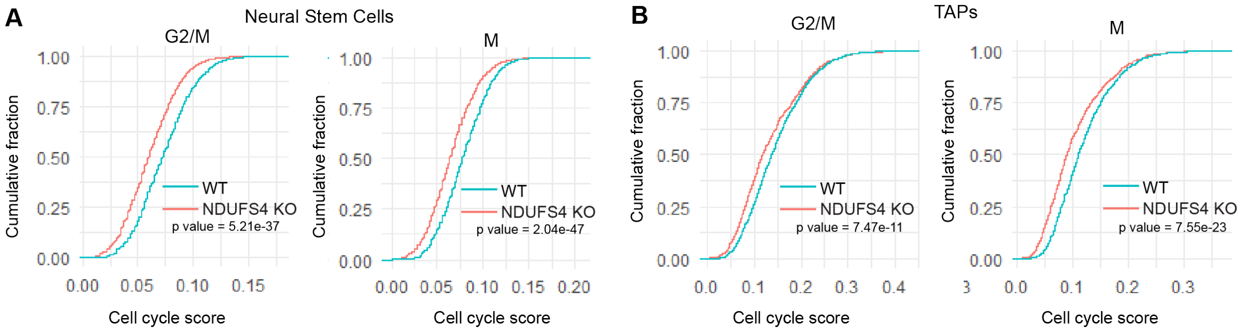

Appendix Figure S1. Cell cycle scoring from scRNA sequencing data of the SVZ of NDUFS4 KO mice display perturbed proliferation.

A, B Comparison of cumulative fractions of cell cycle scores for G2/M and M phases between WT and NDUFS4 KO **(A)** NSCs and **(B)** TAPs.

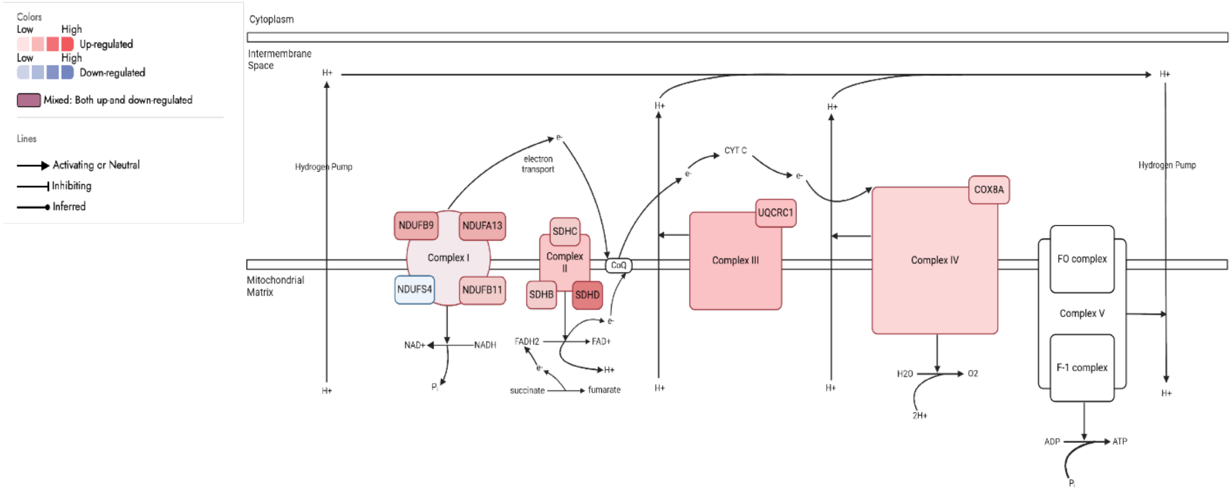

Appendix Figure S2. Increased expression of Complex II-IV subunits in the NDUFS4 KO NSCs. Cartoon Depiction of altered mitochondrial genes associated with the respiratory chain complexes identified from DEGs of NDUFS4 KO NSCs.
