# Supplementary figures and images for: Impaired Complex I dysregulates neural/glial precursors and corpus callosum development revealing postnatal defects in Leigh syndrome mice
Source: EMBO Mol Med. 2025 Dec 22;18(2):677–701. doi: 10.1038/s44321-025-00367-4 (PMC12905379; doi:10.1038/s44321-025-00367-4)

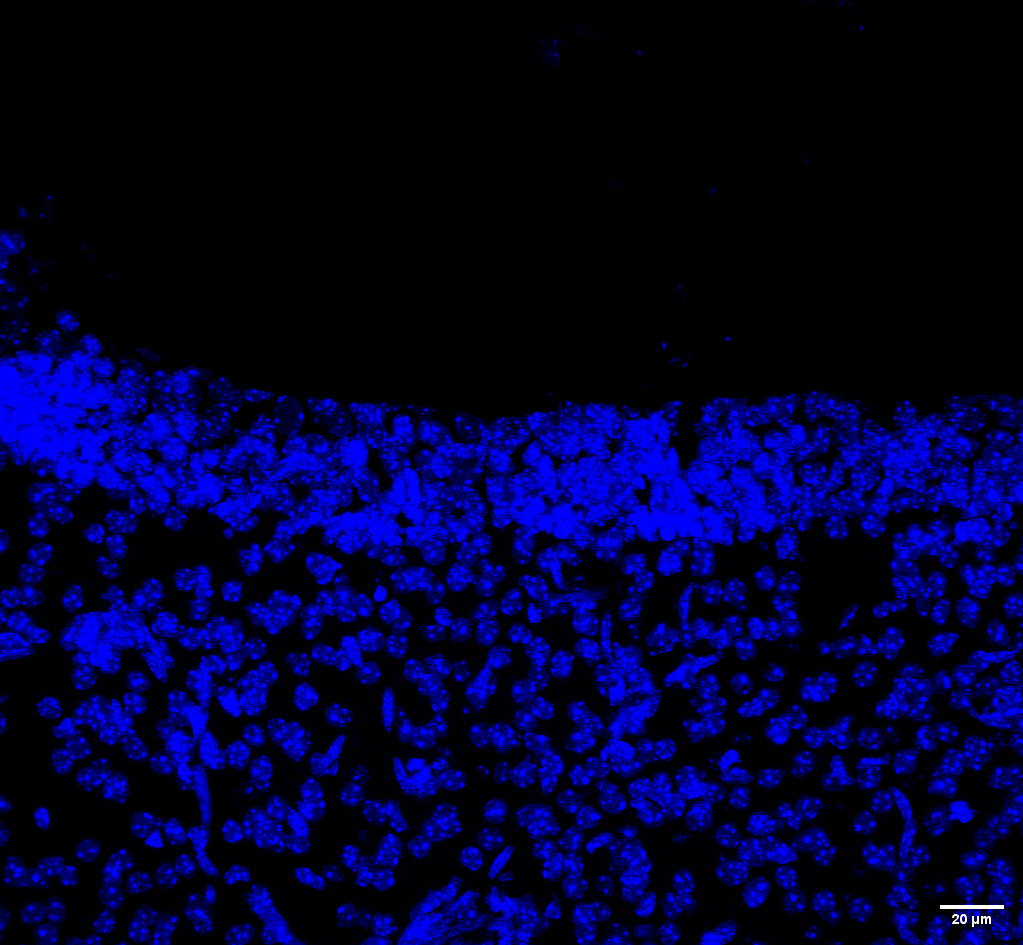

Supplement: Supplementary file 7 — Source data Fig. 2 [file 44321_2025_367_MOESM7_ESM.zip › Figure 2/Figure 2B/NDUFS4 KO_P14_DAPI.tif]

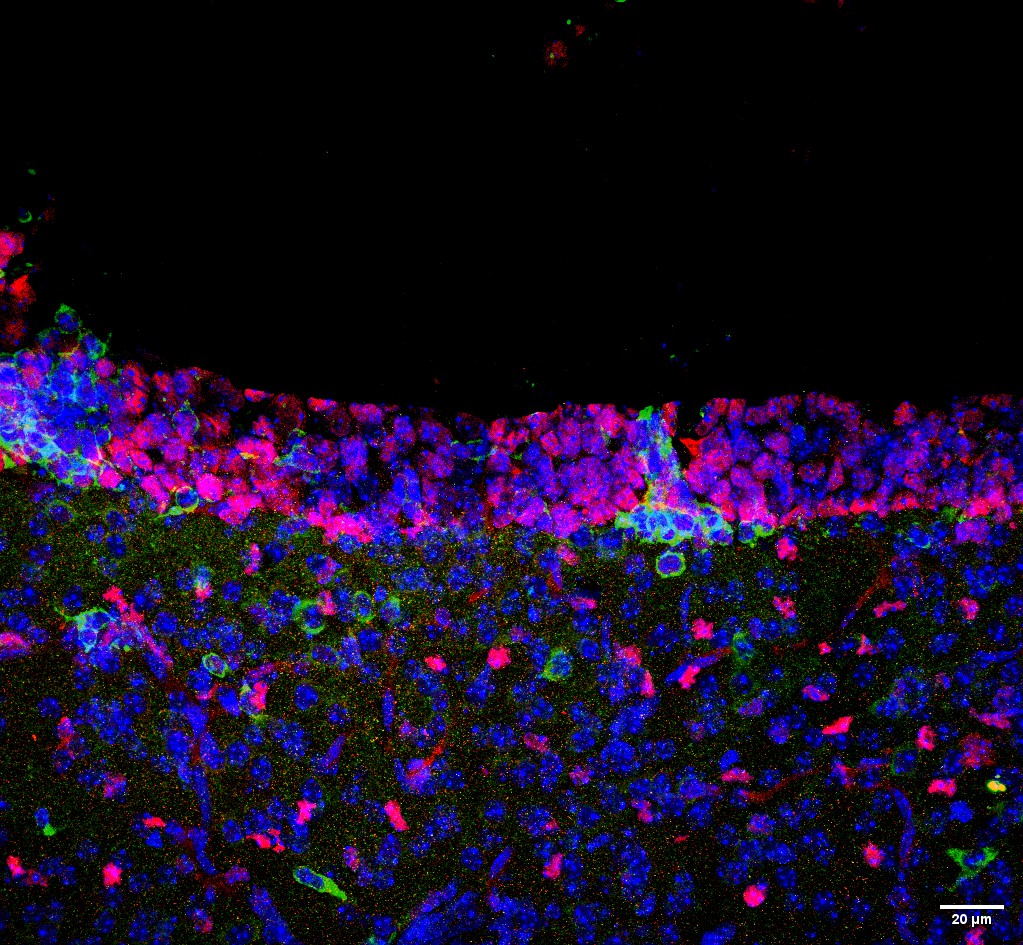

Supplement: Supplementary file 7 — Source data Fig. 2 [file 44321_2025_367_MOESM7_ESM.zip › Figure 2/Figure 2B/NDUFS4 KO_P14_DAPI_SOX2_DCX_Composite.tif]

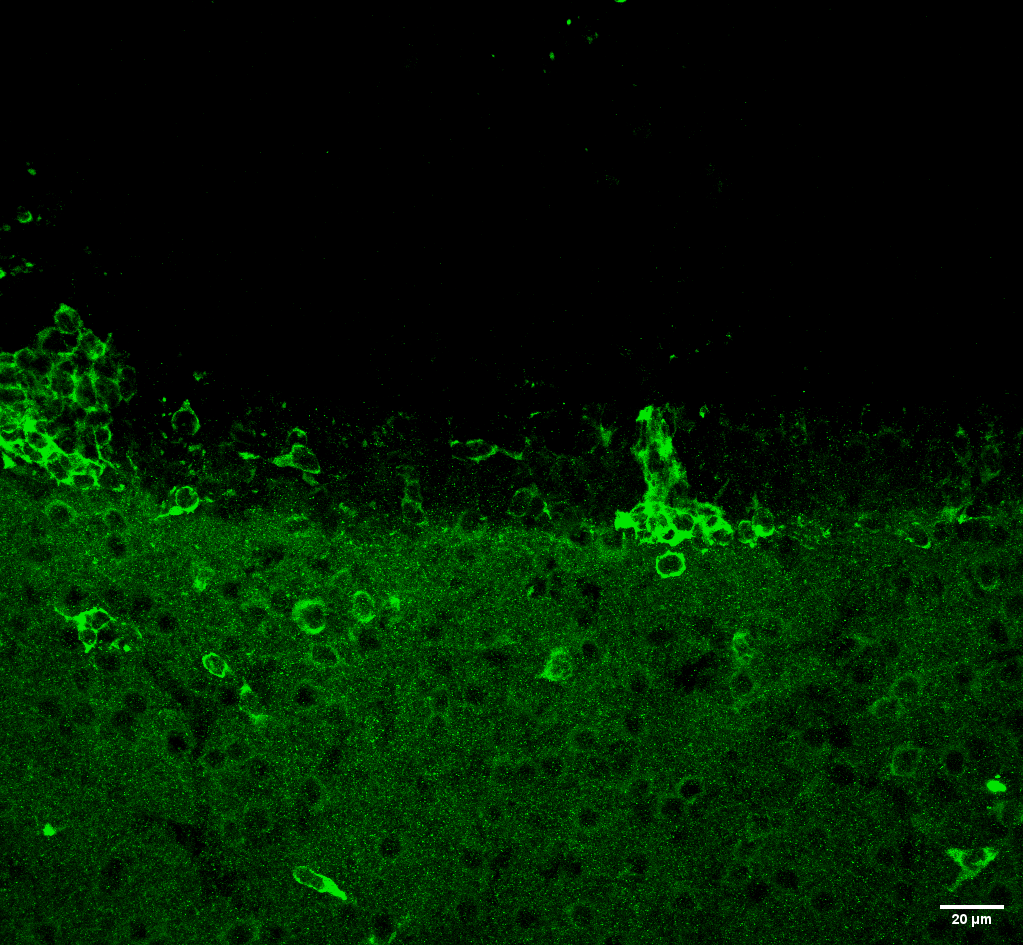

Supplement: Supplementary file 7 — Source data Fig. 2 [file 44321_2025_367_MOESM7_ESM.zip › Figure 2/Figure 2B/NDUFS4 KO_P14_DCX.tif]

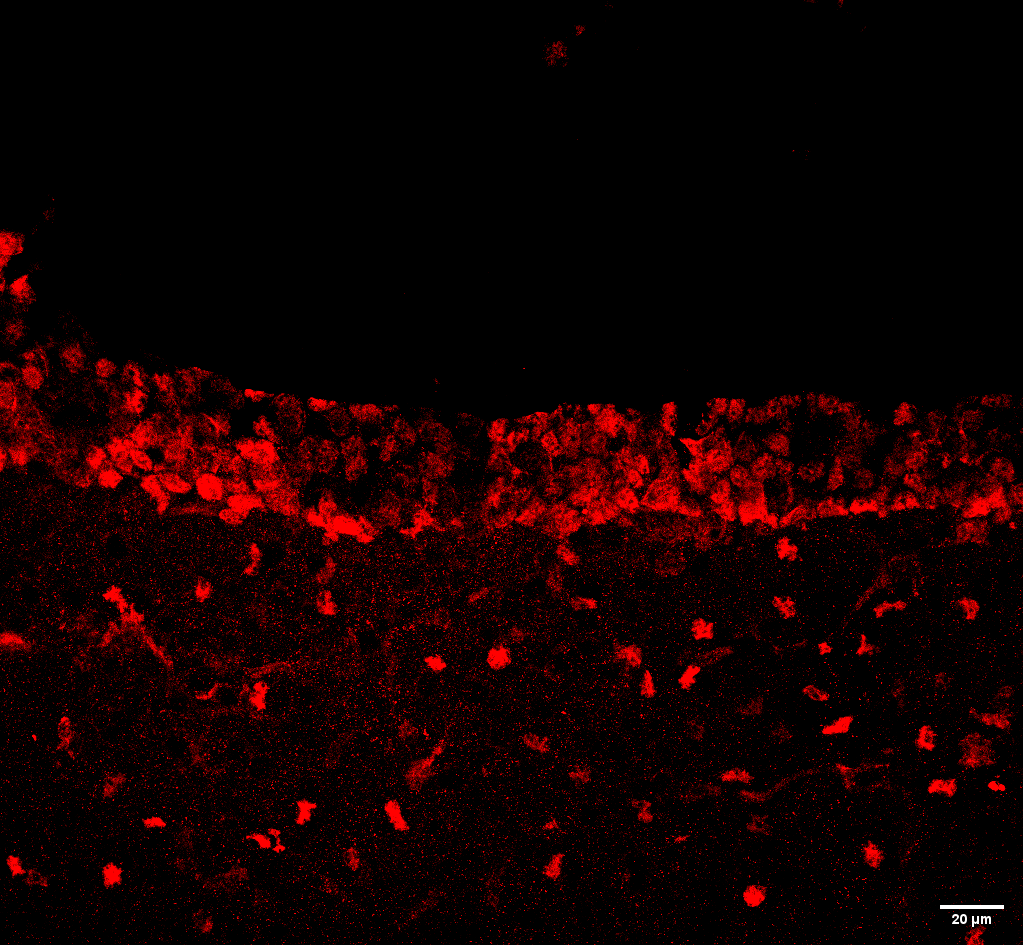

Supplement: Supplementary file 7 — Source data Fig. 2 [file 44321_2025_367_MOESM7_ESM.zip › Figure 2/Figure 2B/NDUFS4 KO_P14_SOX2.tif]

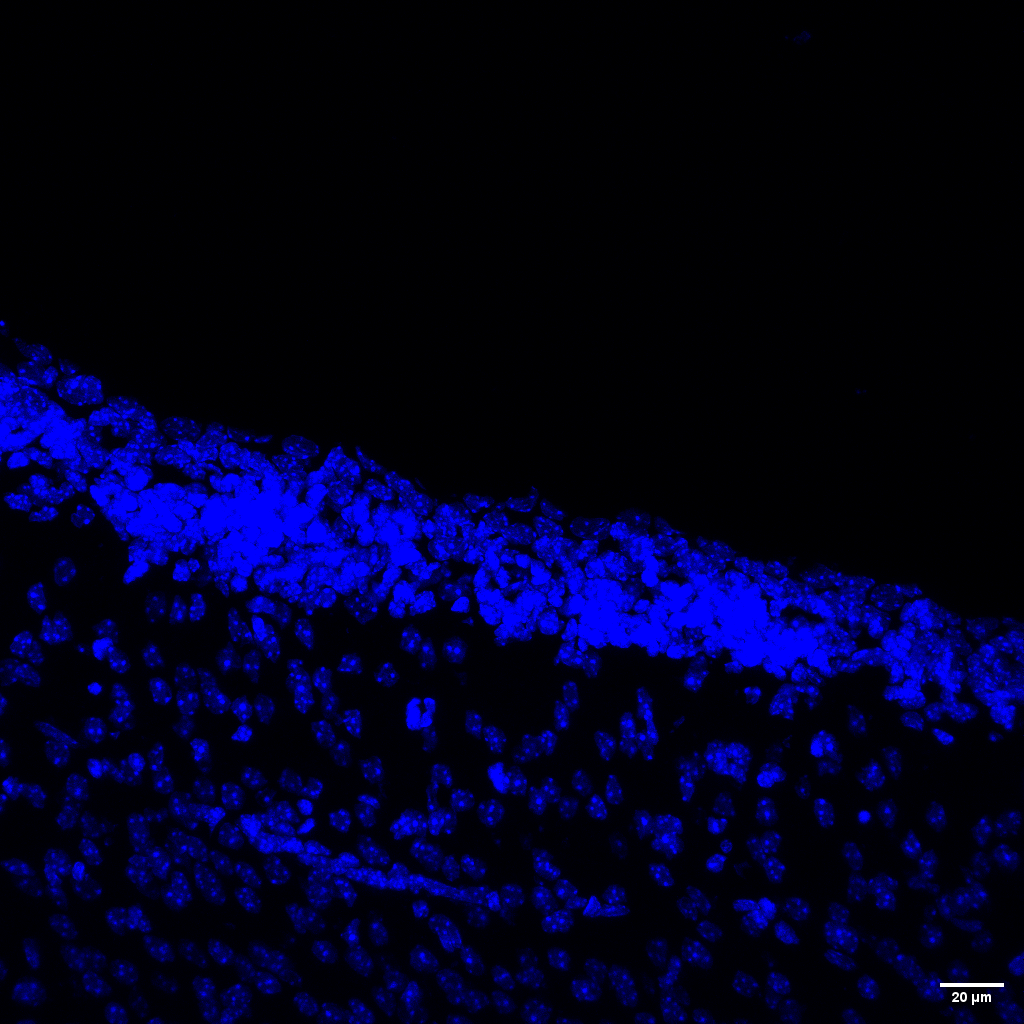

Supplement: Supplementary file 7 — Source data Fig. 2 [file 44321_2025_367_MOESM7_ESM.zip › Figure 2/Figure 2B/WT_P14_DAPI.tif]

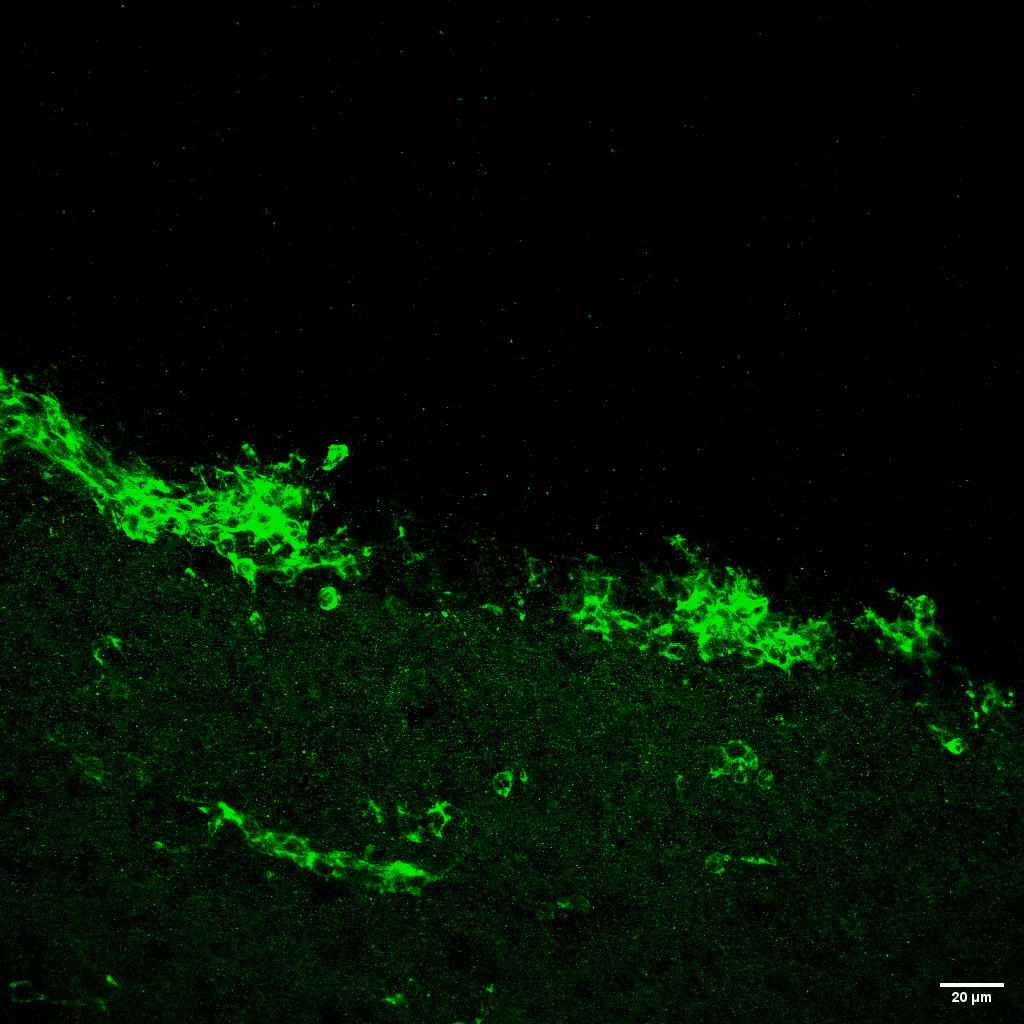

Supplement: Supplementary file 7 — Source data Fig. 2 [file 44321_2025_367_MOESM7_ESM.zip › Figure 2/Figure 2B/WT_P14_DCX.tif]

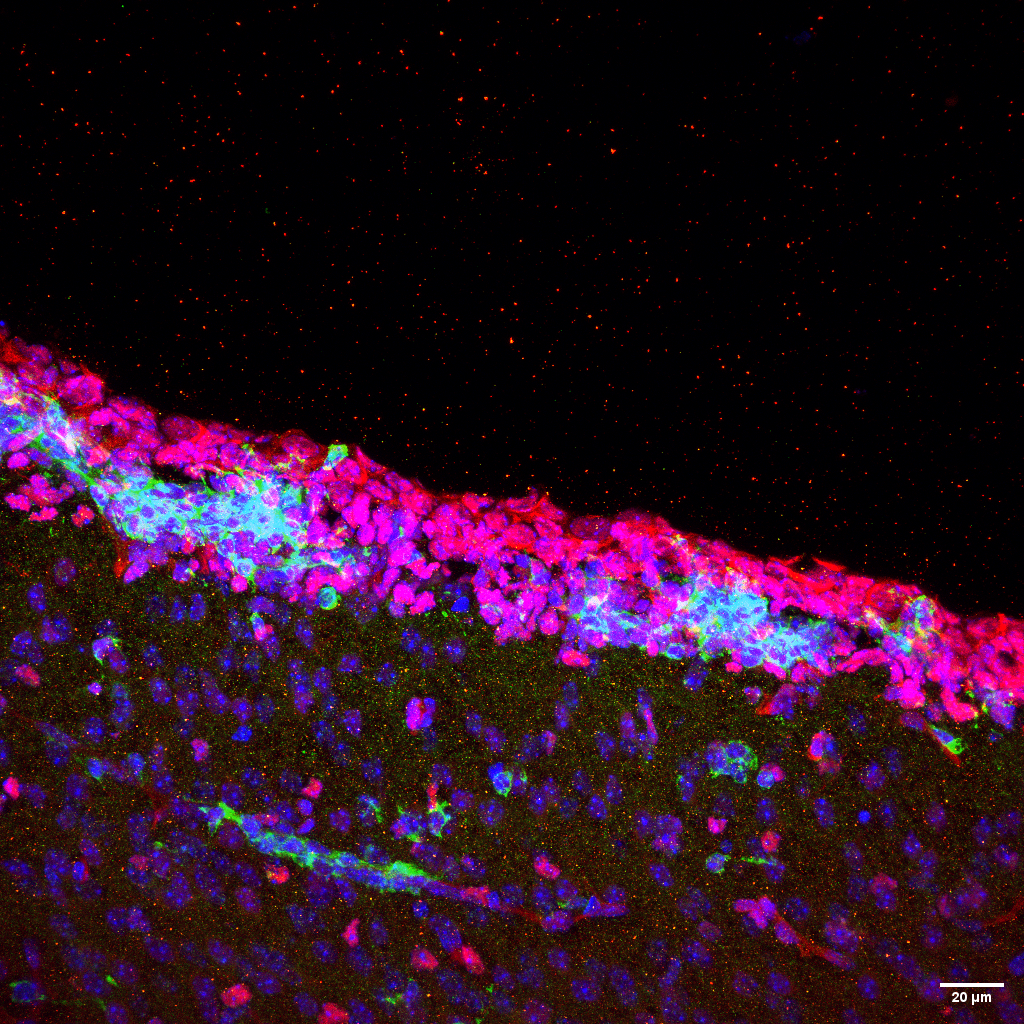

Supplement: Supplementary file 7 — Source data Fig. 2 [file 44321_2025_367_MOESM7_ESM.zip › Figure 2/Figure 2B/WT_P14_SOX2 _DCX_DAPI_Composite.tif]

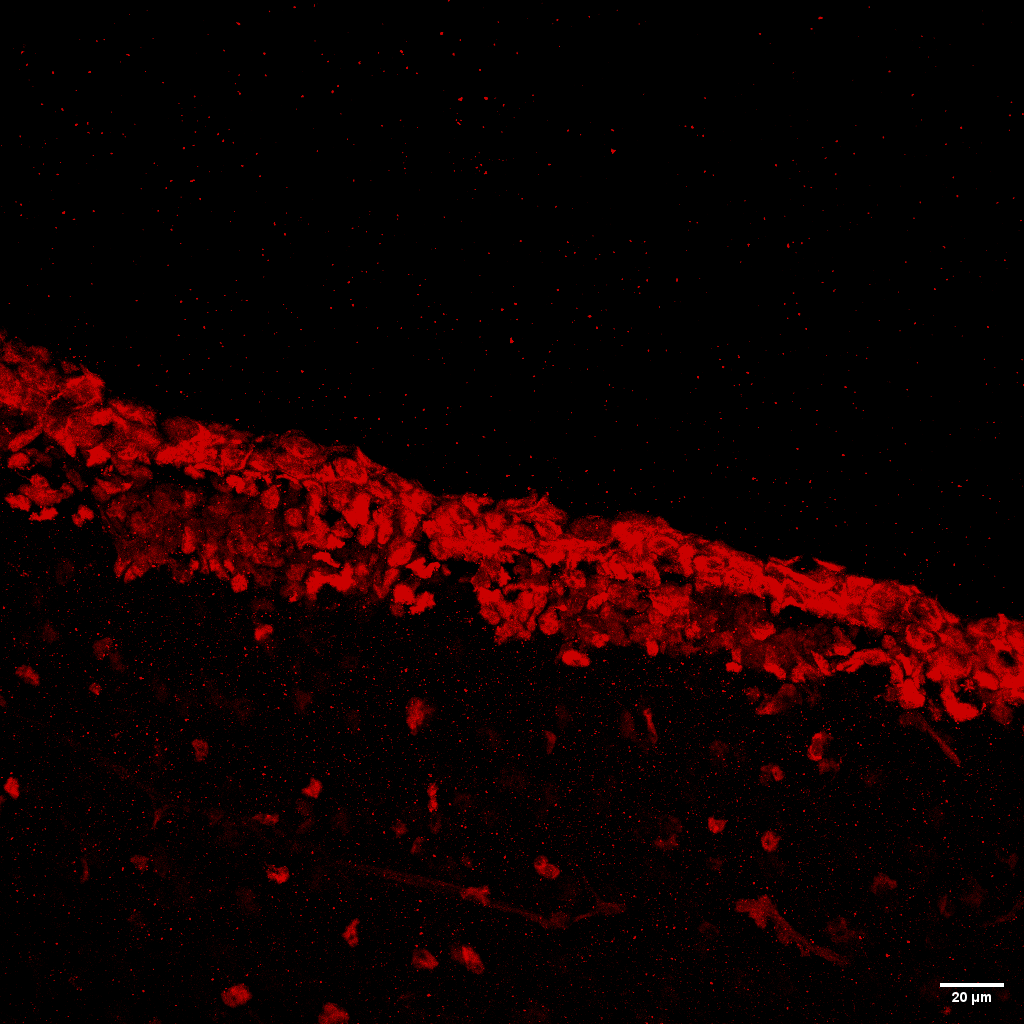

Supplement: Supplementary file 7 — Source data Fig. 2 [file 44321_2025_367_MOESM7_ESM.zip › Figure 2/Figure 2B/WT_P14_SOX2.tif]

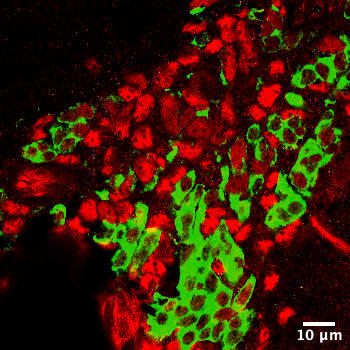

Supplement: Supplementary file 7 — Source data Fig. 2 [file 44321_2025_367_MOESM7_ESM.zip › Figure 2/Figure 2E/NDUFS4 KO_P24_Composite.tif]

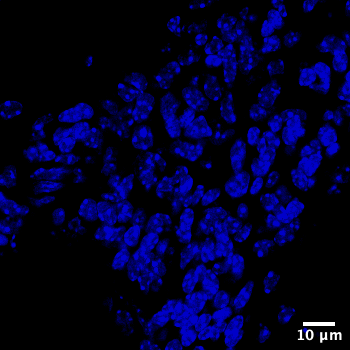

Supplement: Supplementary file 7 — Source data Fig. 2 [file 44321_2025_367_MOESM7_ESM.zip › Figure 2/Figure 2E/NDUFS4 KO_P24_DAPI.tif]

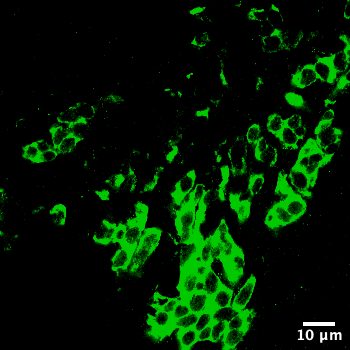

Supplement: Supplementary file 7 — Source data Fig. 2 [file 44321_2025_367_MOESM7_ESM.zip › Figure 2/Figure 2E/NDUFS4 KO_P24_DCX.tif]

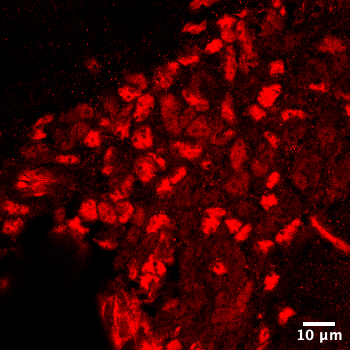

Supplement: Supplementary file 7 — Source data Fig. 2 [file 44321_2025_367_MOESM7_ESM.zip › Figure 2/Figure 2E/NDUFS4 KO_P24_SOX2.tif]

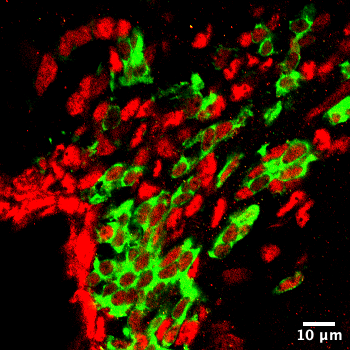

Supplement: Supplementary file 7 — Source data Fig. 2 [file 44321_2025_367_MOESM7_ESM.zip › Figure 2/Figure 2E/WT_P24_Composite.tif]

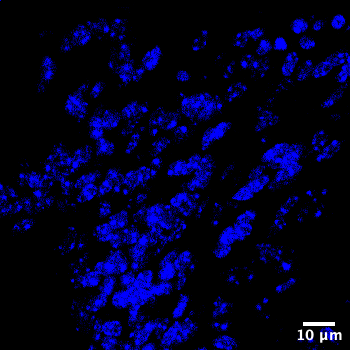

Supplement: Supplementary file 7 — Source data Fig. 2 [file 44321_2025_367_MOESM7_ESM.zip › Figure 2/Figure 2E/WT_P24_DAPI.tif]

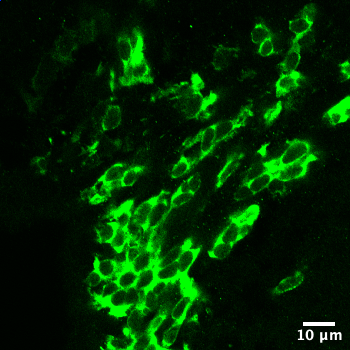

Supplement: Supplementary file 7 — Source data Fig. 2 [file 44321_2025_367_MOESM7_ESM.zip › Figure 2/Figure 2E/WT_P24_DCX.tif]

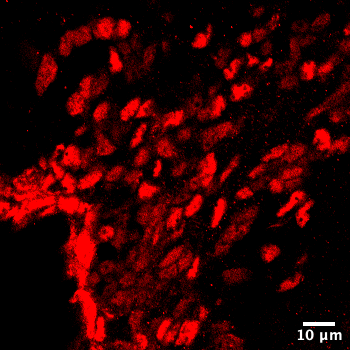

Supplement: Supplementary file 7 — Source data Fig. 2 [file 44321_2025_367_MOESM7_ESM.zip › Figure 2/Figure 2E/WT_P24_SOX2.tif]

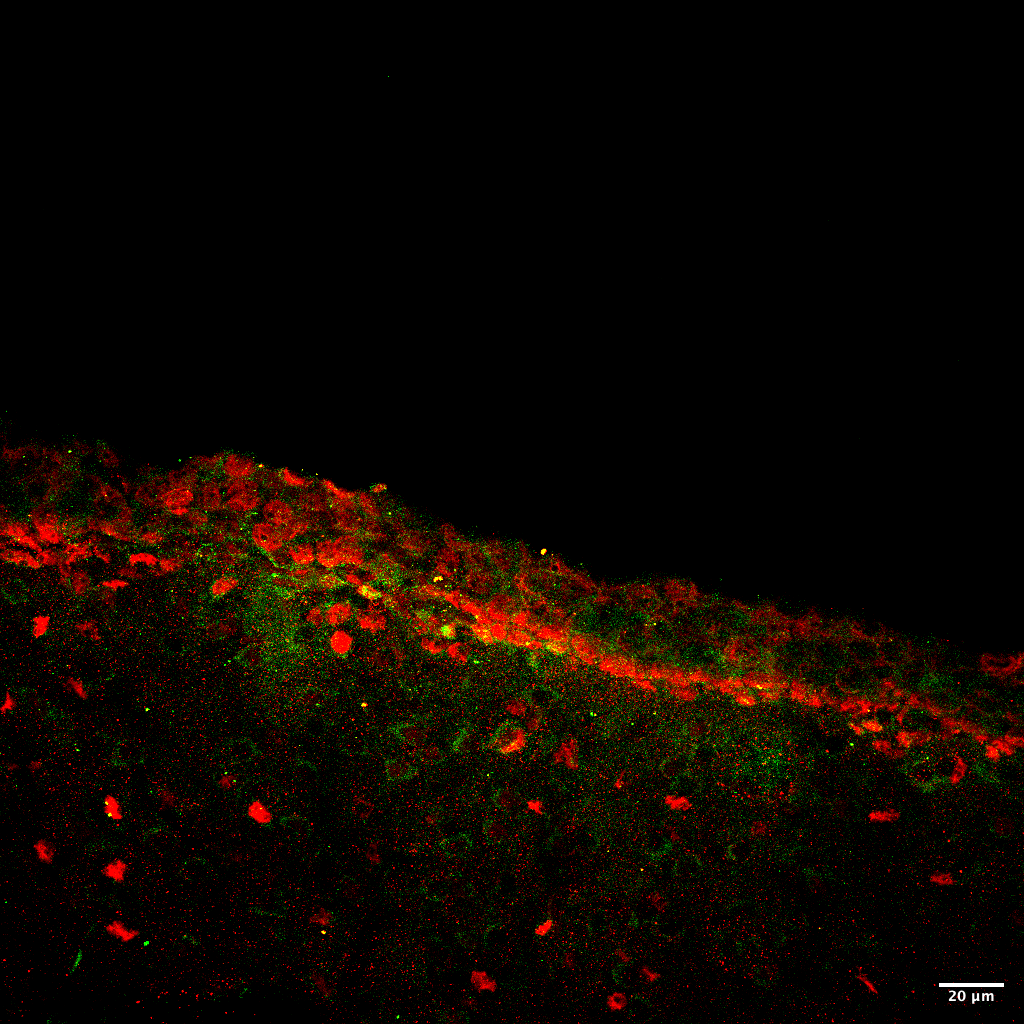

Supplement: Supplementary file 8 — Source data Fig. 3 [file 44321_2025_367_MOESM8_ESM.zip › Figure 3/Figure 3D/NDUFS4 KO_P14_Composite.tif]

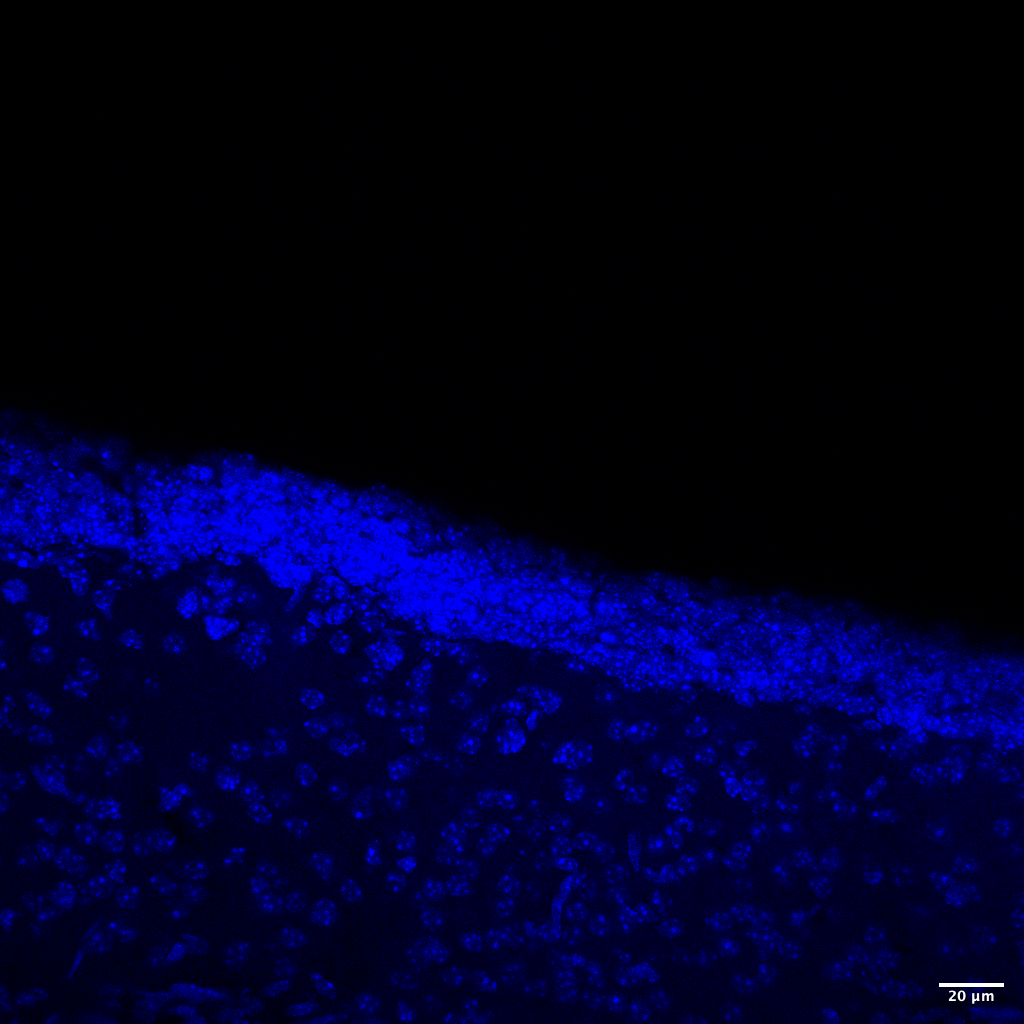

Supplement: Supplementary file 8 — Source data Fig. 3 [file 44321_2025_367_MOESM8_ESM.zip › Figure 3/Figure 3D/NDUFS4 KO_P14_DAPI.tif]

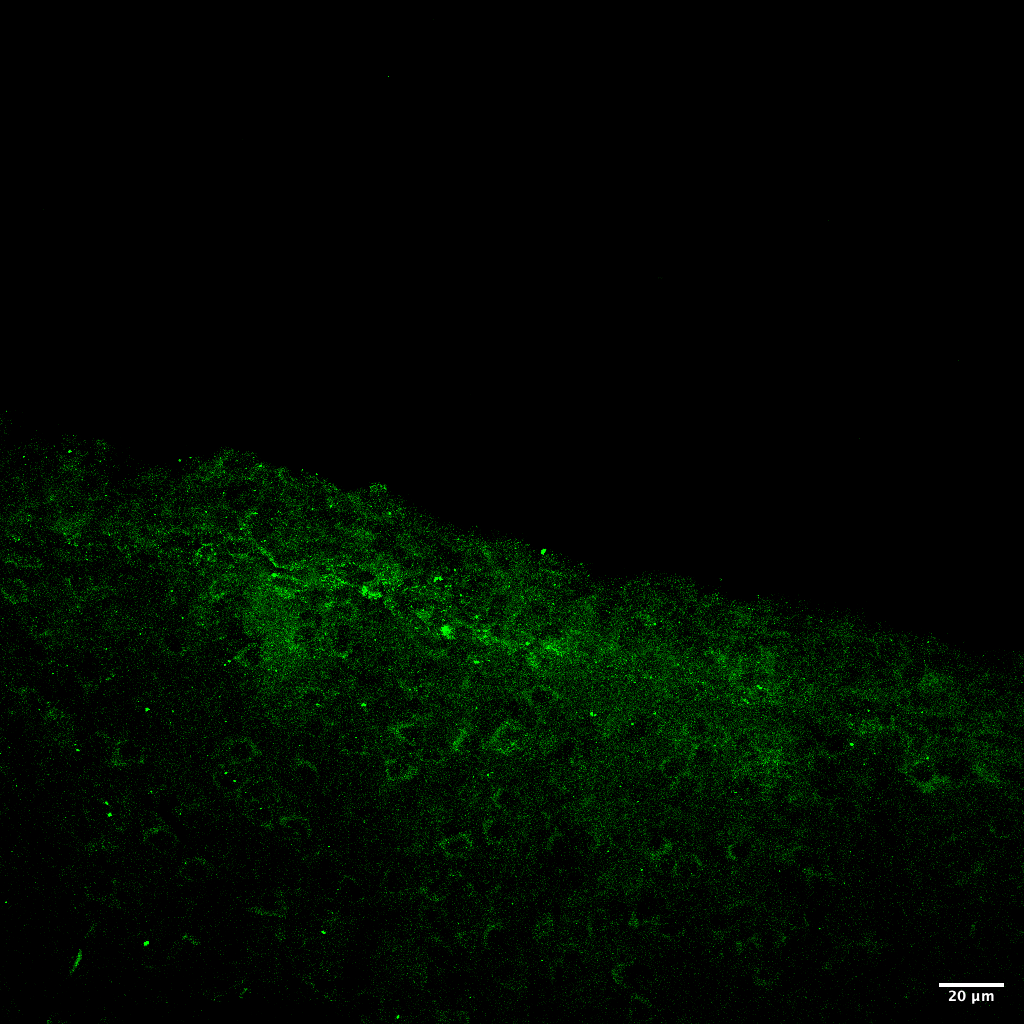

Supplement: Supplementary file 8 — Source data Fig. 3 [file 44321_2025_367_MOESM8_ESM.zip › Figure 3/Figure 3D/NDUFS4 KO_P14_Ki67.tif]

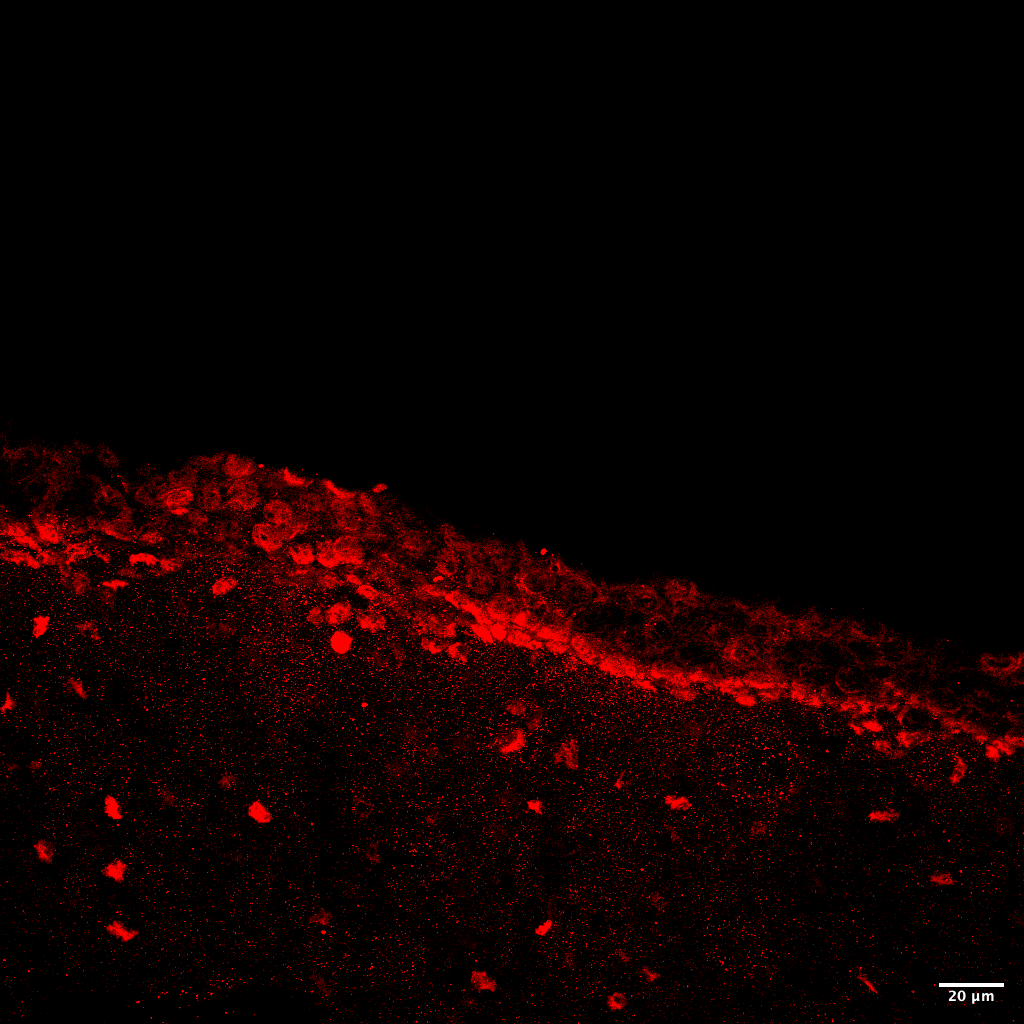

Supplement: Supplementary file 8 — Source data Fig. 3 [file 44321_2025_367_MOESM8_ESM.zip › Figure 3/Figure 3D/NDUFS4 KO_P14_SOX2.tif]

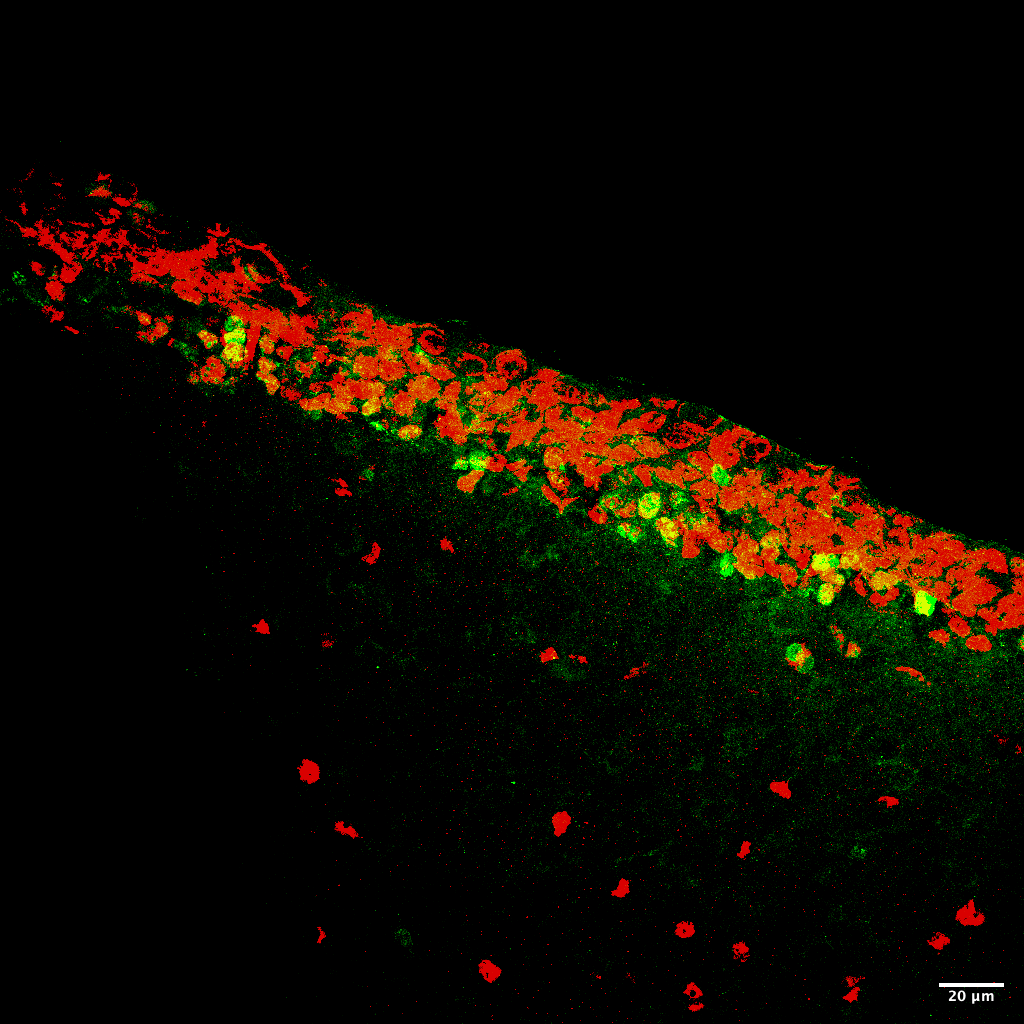

Supplement: Supplementary file 8 — Source data Fig. 3 [file 44321_2025_367_MOESM8_ESM.zip › Figure 3/Figure 3D/WT_P14_Composite.tif]

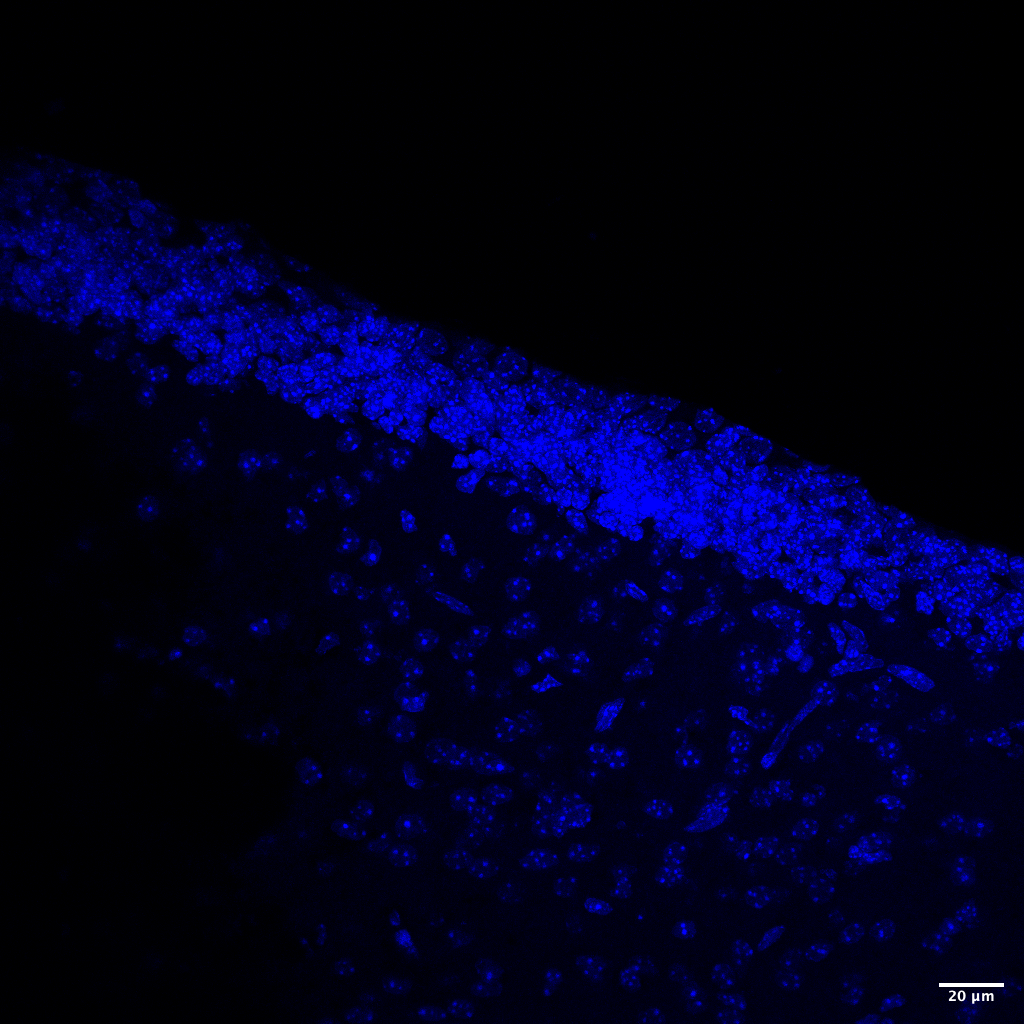

Supplement: Supplementary file 8 — Source data Fig. 3 [file 44321_2025_367_MOESM8_ESM.zip › Figure 3/Figure 3D/WT_P14_DAPI.tif]

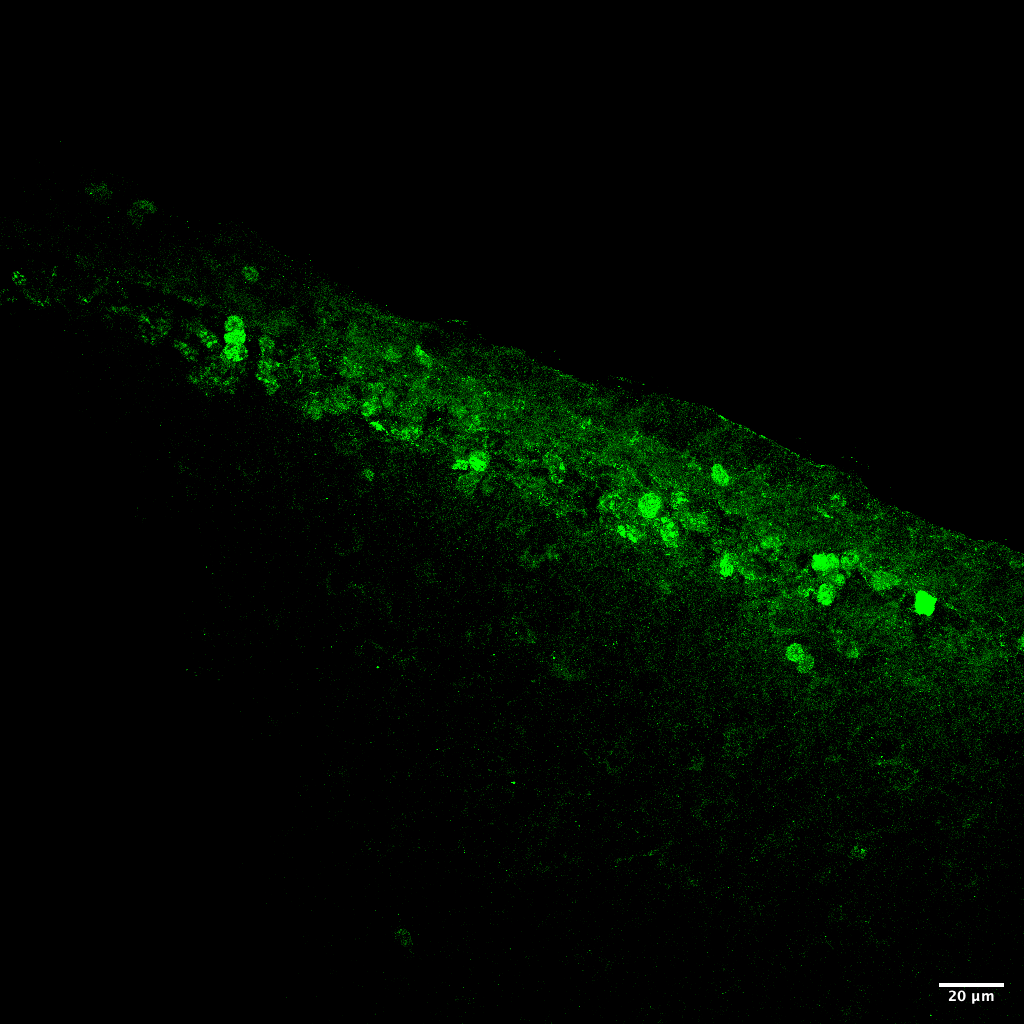

Supplement: Supplementary file 8 — Source data Fig. 3 [file 44321_2025_367_MOESM8_ESM.zip › Figure 3/Figure 3D/WT_P14_Ki67.tif]

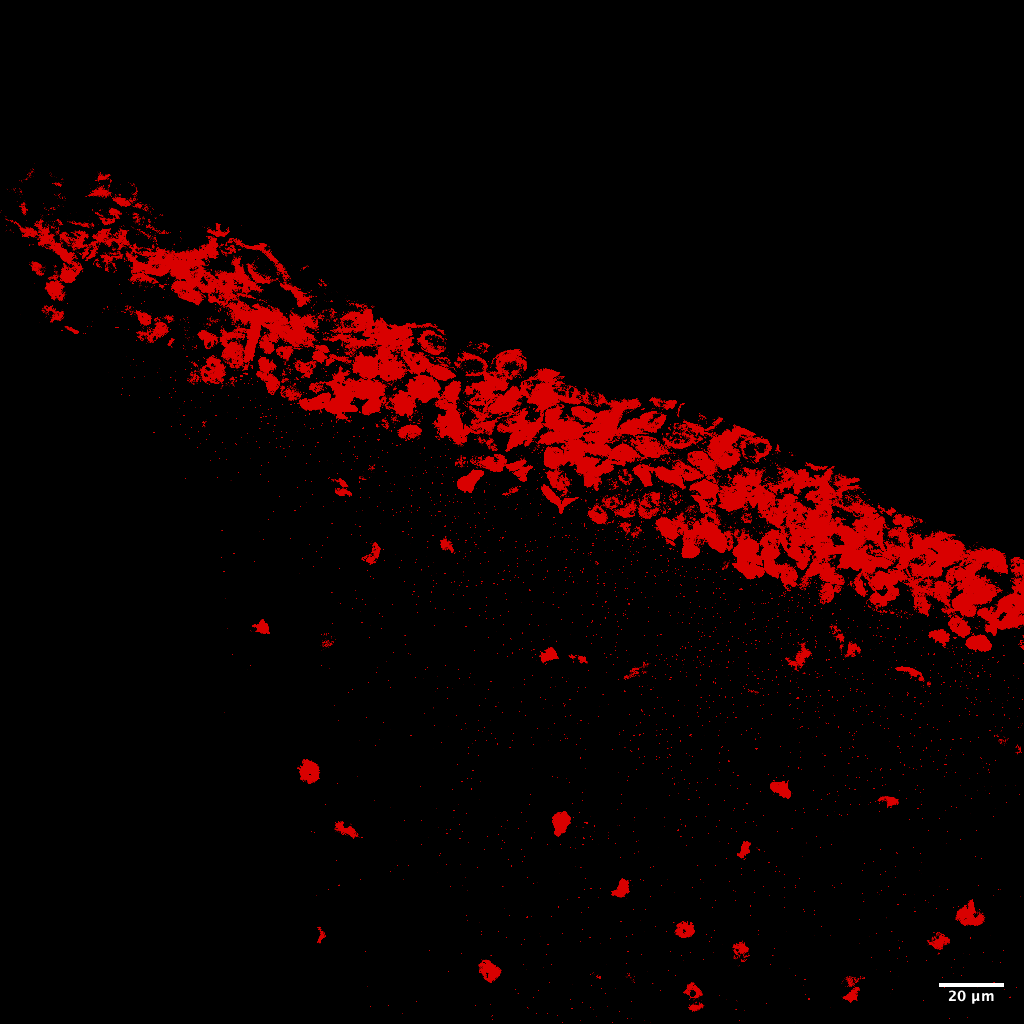

Supplement: Supplementary file 8 — Source data Fig. 3 [file 44321_2025_367_MOESM8_ESM.zip › Figure 3/Figure 3D/WT_P14_SOX2.tif]

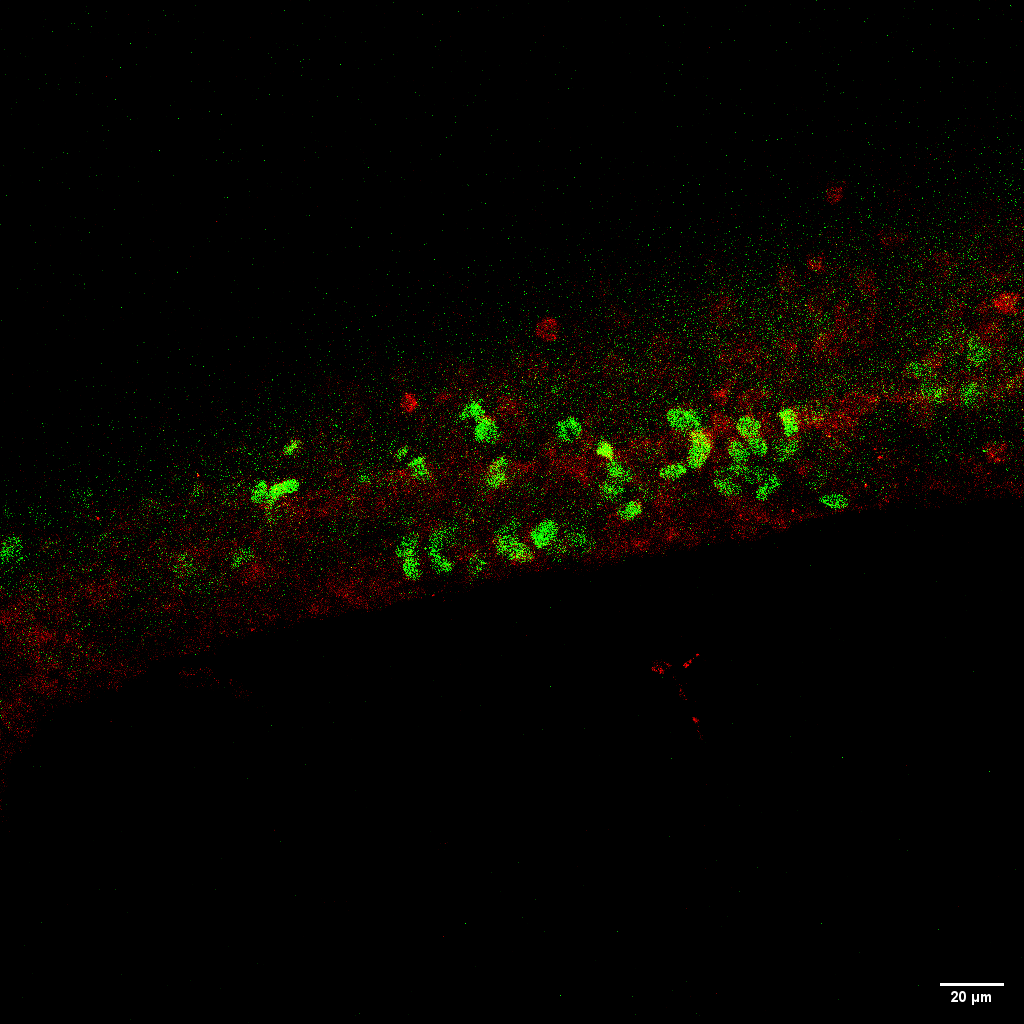

Supplement: Supplementary file 11 — Source data Fig. 6 [file 44321_2025_367_MOESM11_ESM.zip › Figure 6/Figure 6B/NDUFS4 KO_P14_Composite.tif]

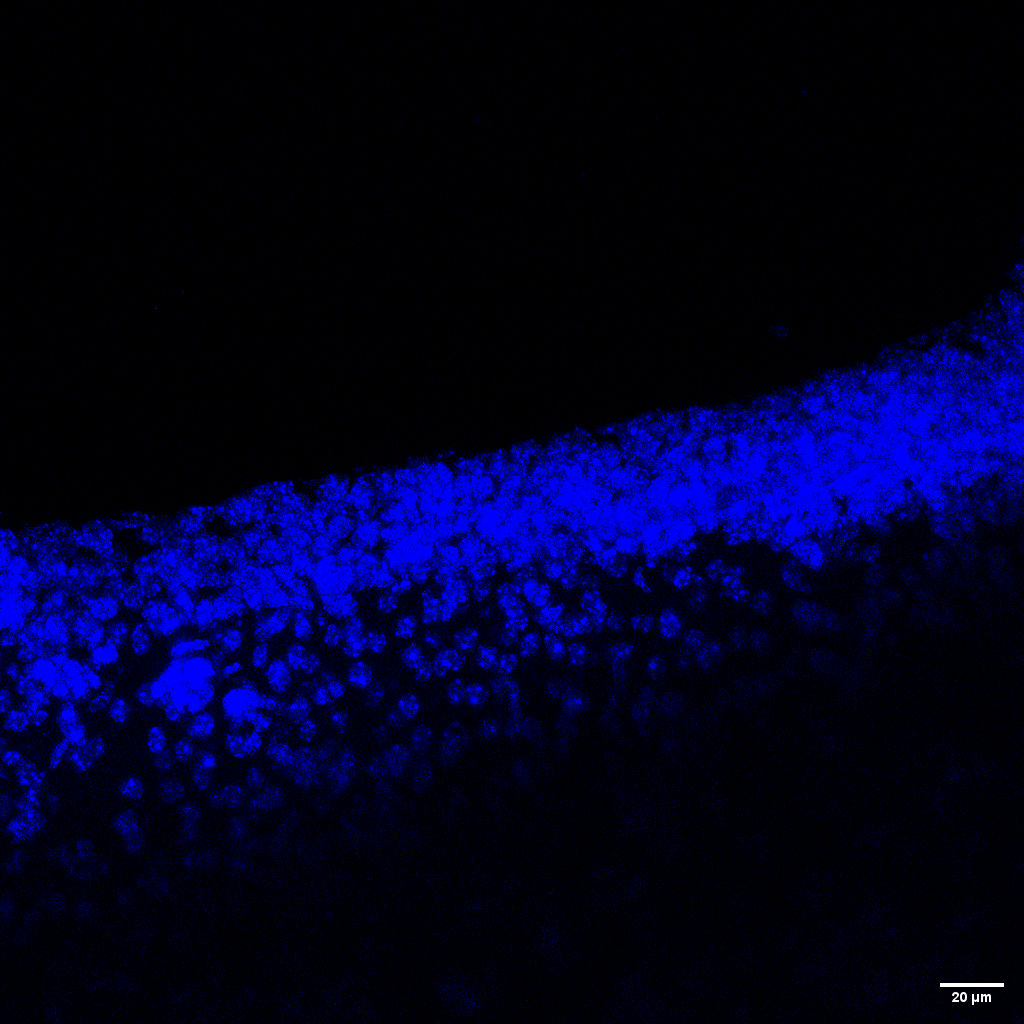

Supplement: Supplementary file 11 — Source data Fig. 6 [file 44321_2025_367_MOESM11_ESM.zip › Figure 6/Figure 6B/NDUFS4 KO_P14_DAPI.tif]

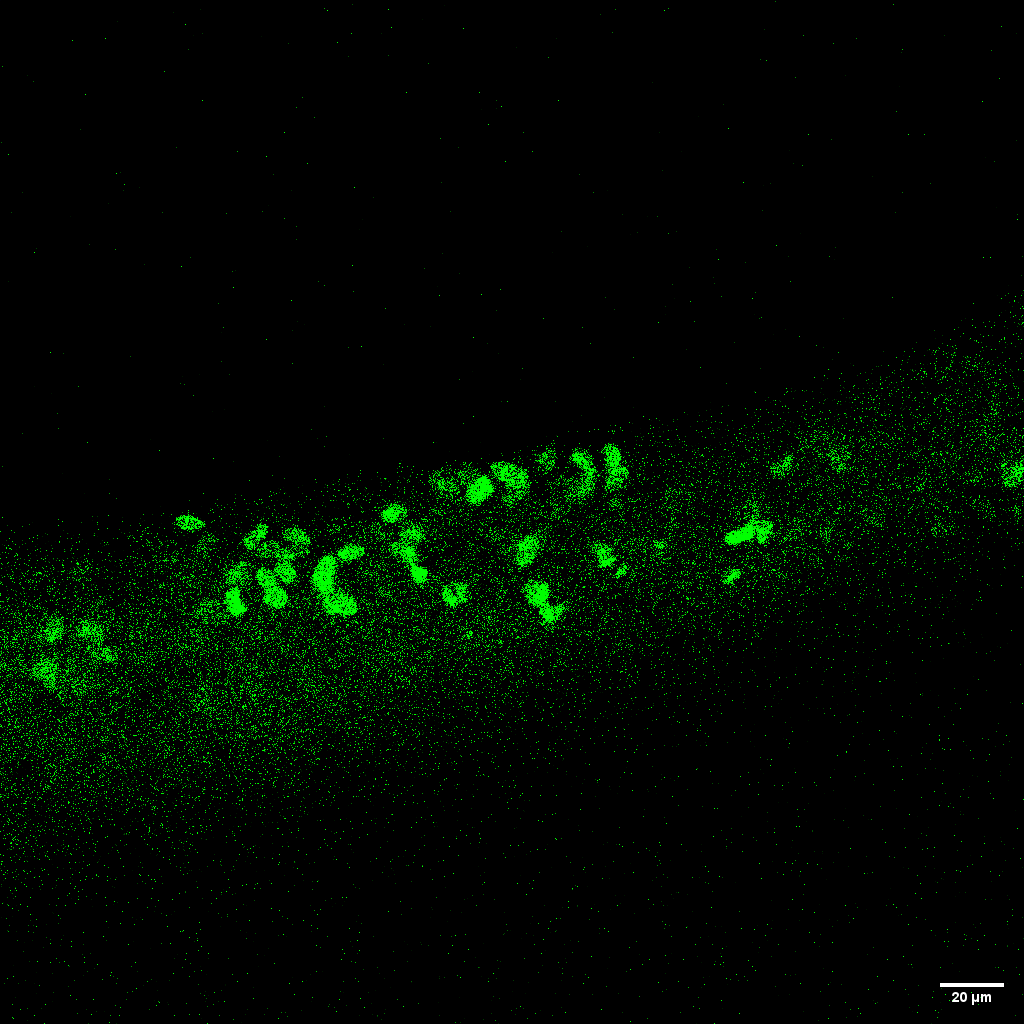

Supplement: Supplementary file 11 — Source data Fig. 6 [file 44321_2025_367_MOESM11_ESM.zip › Figure 6/Figure 6B/NDUFS4 KO_P14_MASH1.tif]

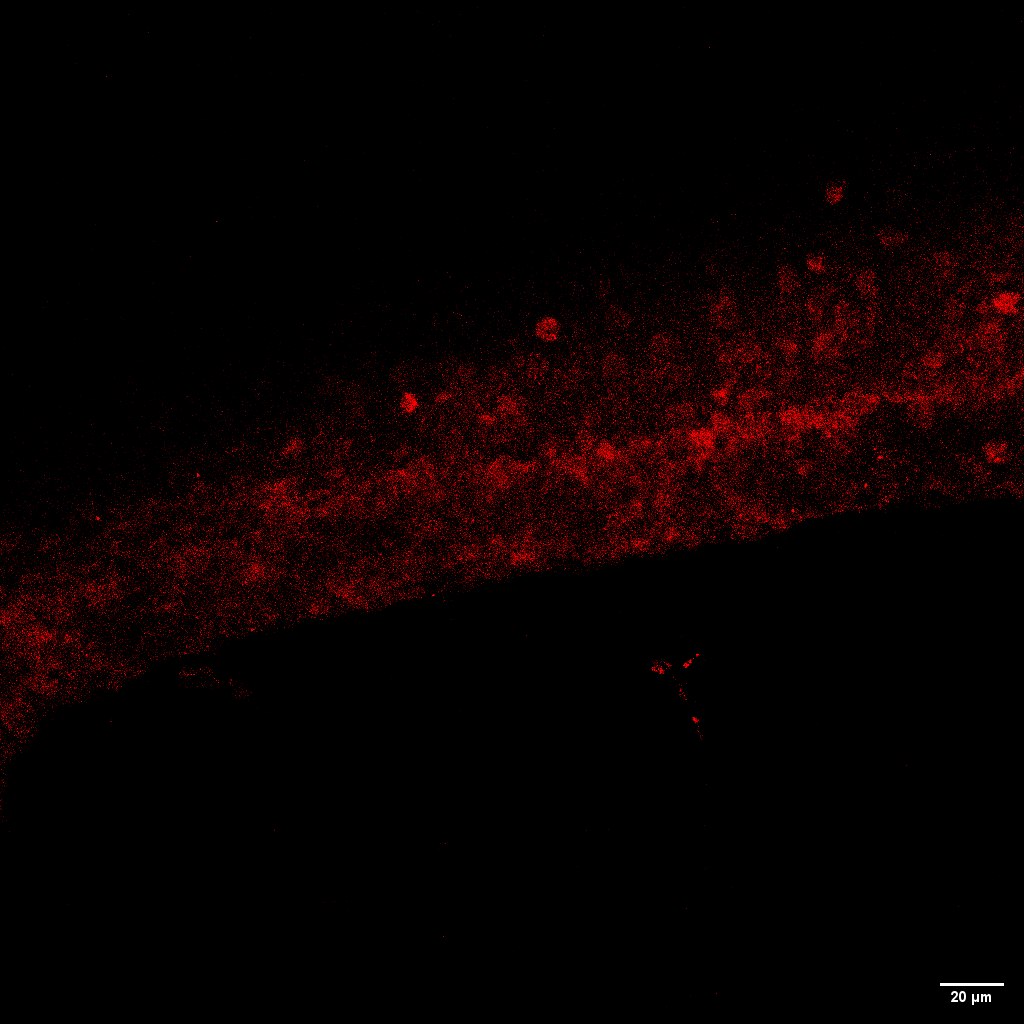

Supplement: Supplementary file 11 — Source data Fig. 6 [file 44321_2025_367_MOESM11_ESM.zip › Figure 6/Figure 6B/NDUFS4 KO_P14_Nkx.tif]

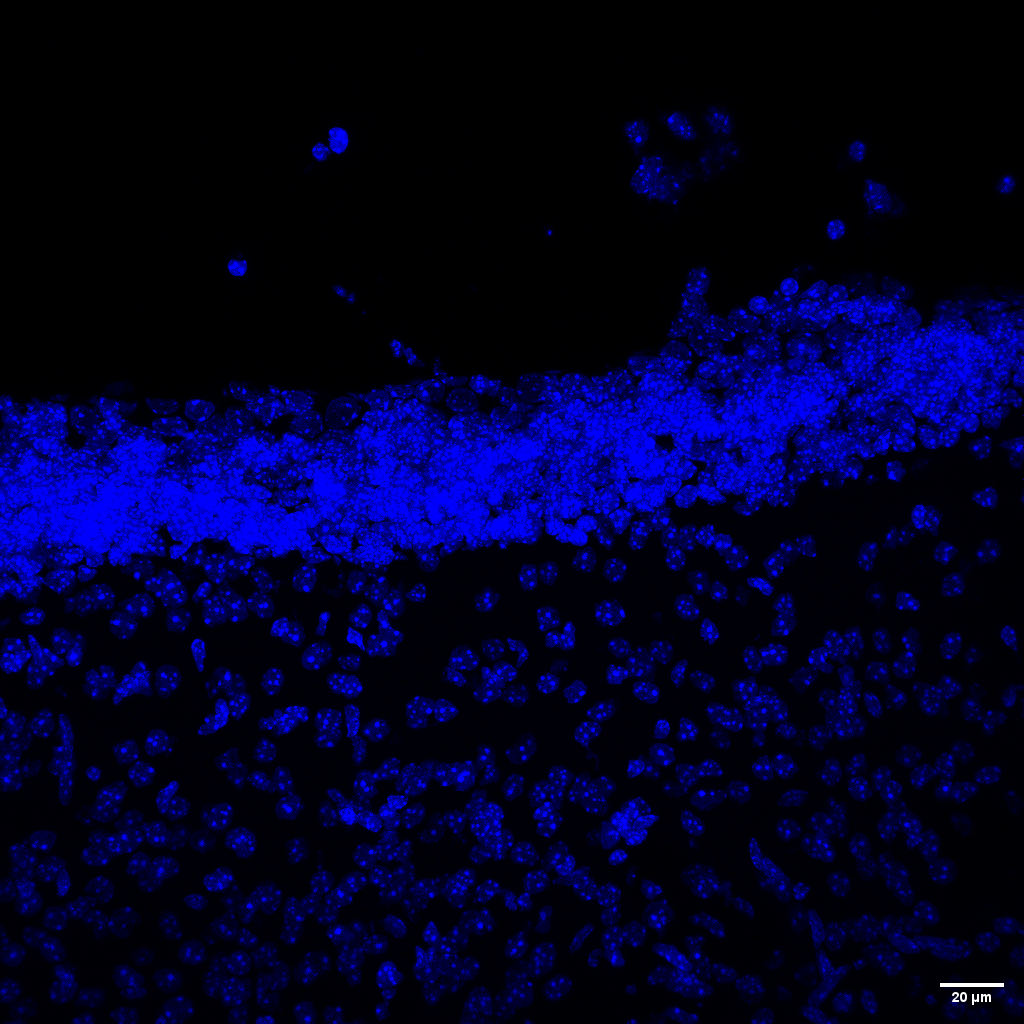

Supplement: Supplementary file 11 — Source data Fig. 6 [file 44321_2025_367_MOESM11_ESM.zip › Figure 6/Figure 6B/WT_P14_DAPI.tif]

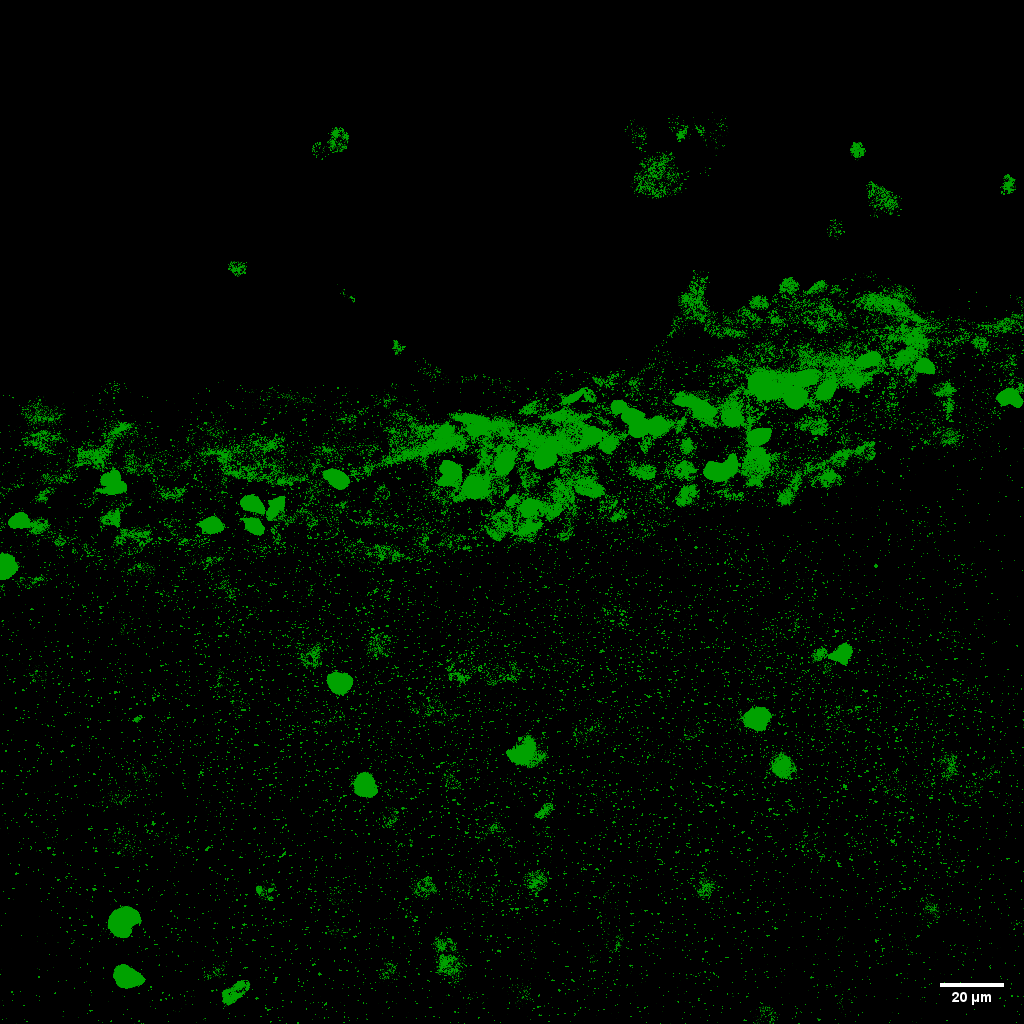

Supplement: Supplementary file 11 — Source data Fig. 6 [file 44321_2025_367_MOESM11_ESM.zip › Figure 6/Figure 6B/WT_P14_MASH1.tif]

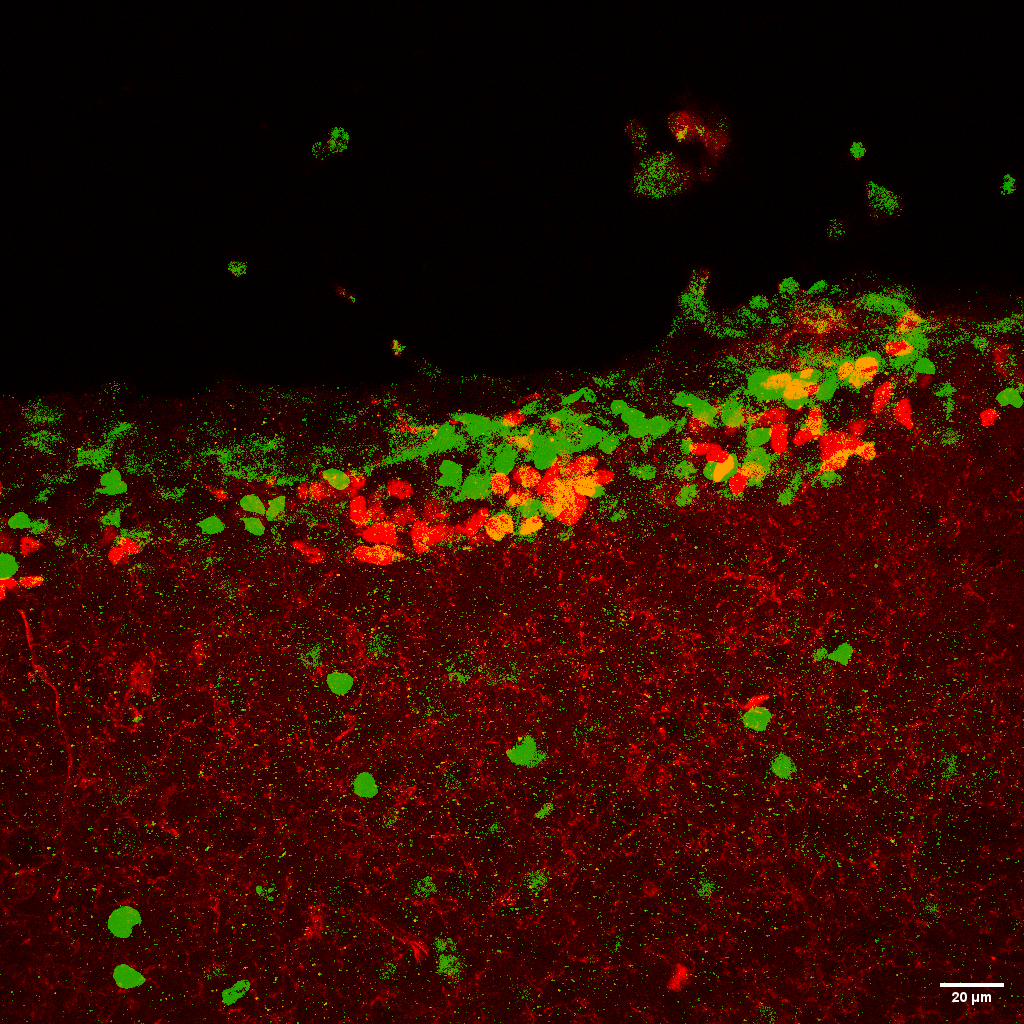

Supplement: Supplementary file 11 — Source data Fig. 6 [file 44321_2025_367_MOESM11_ESM.zip › Figure 6/Figure 6B/WT_P14_MASH1_Nkx_Composite.tif]

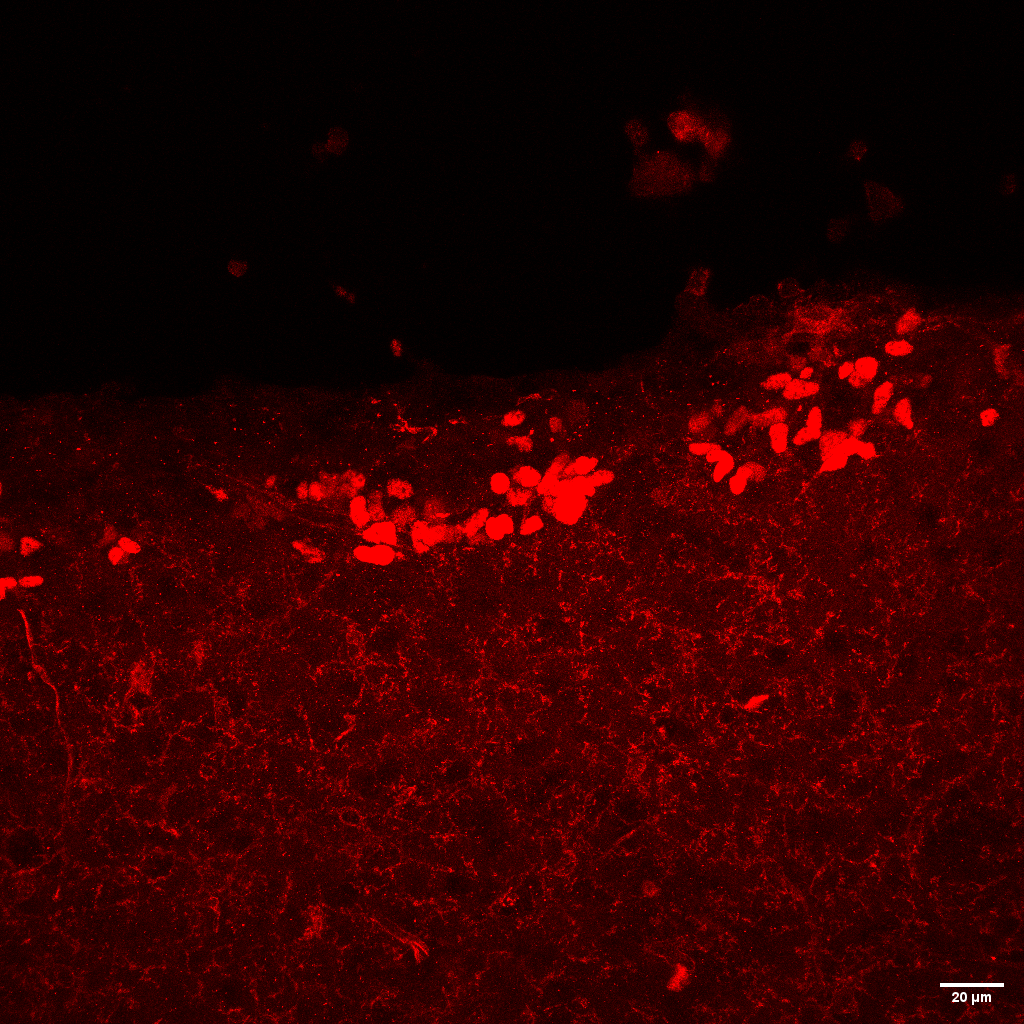

Supplement: Supplementary file 11 — Source data Fig. 6 [file 44321_2025_367_MOESM11_ESM.zip › Figure 6/Figure 6B/WT_P14_Nkx.tif]

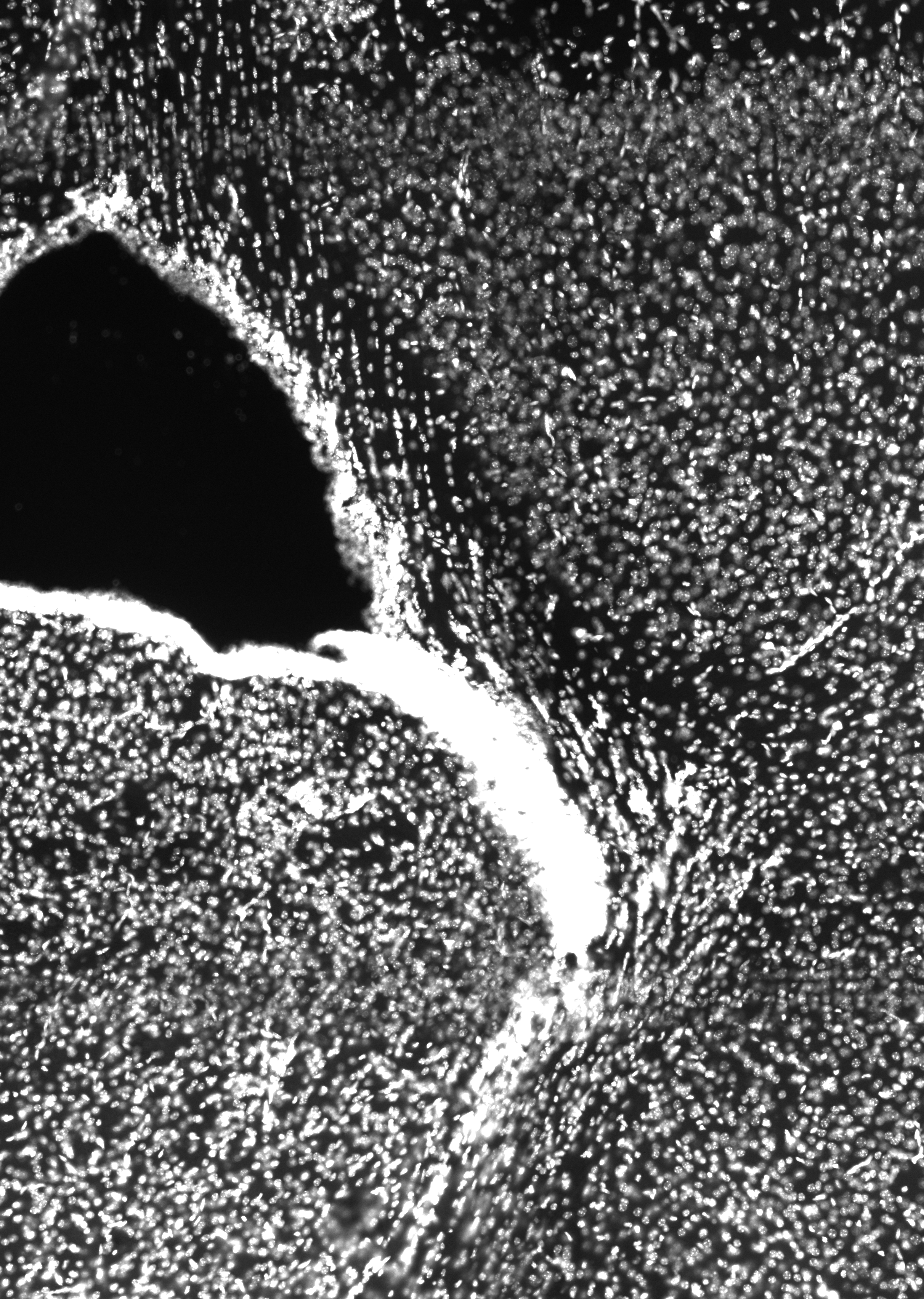

Supplement: Supplementary file 12 — Source data Fig. 7 [file 44321_2025_367_MOESM12_ESM.zip › Figure 7_New/Figure 7E/NDUFS4 KO_CC thickness_P14.tif]

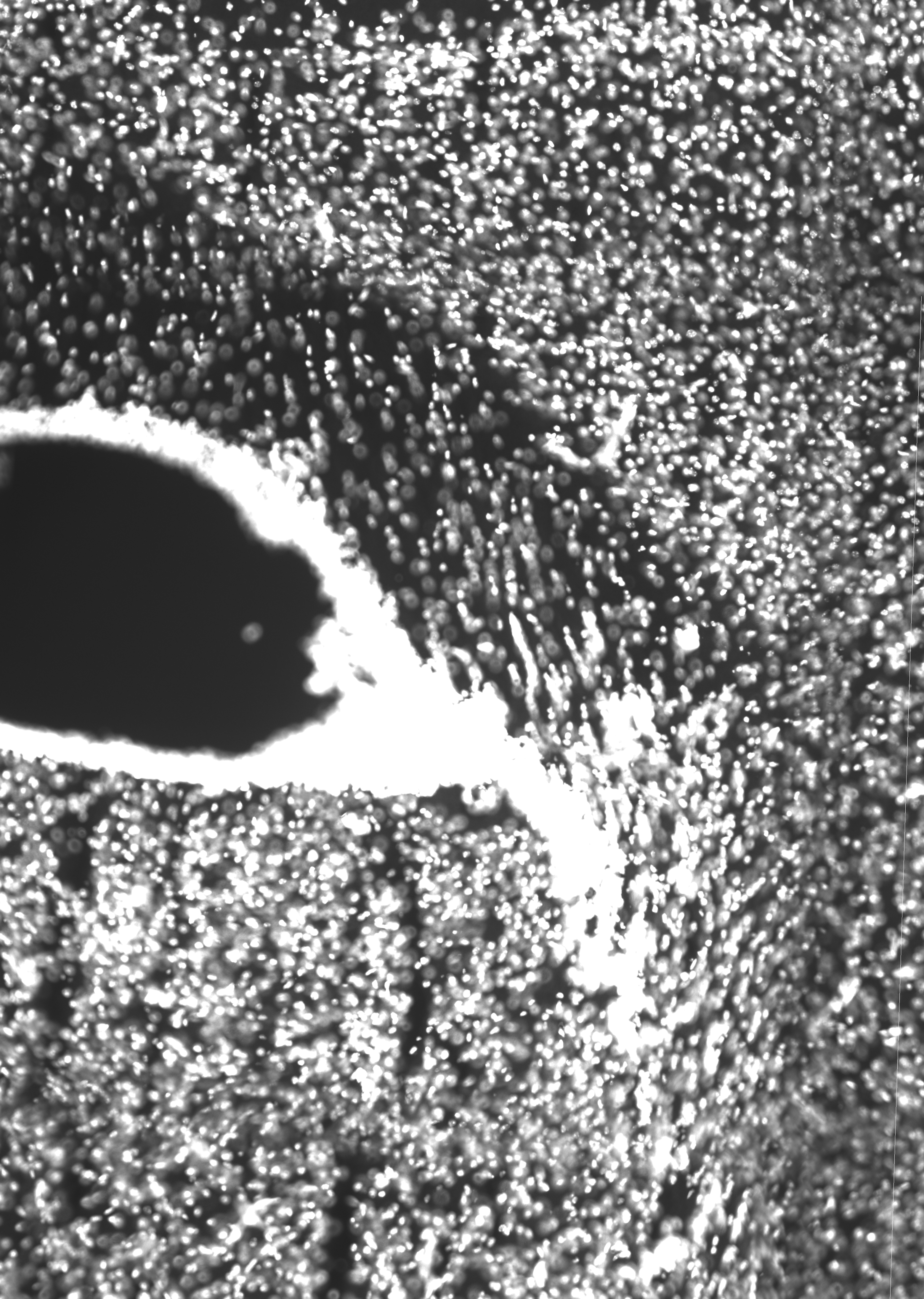

Supplement: Supplementary file 12 — Source data Fig. 7 [file 44321_2025_367_MOESM12_ESM.zip › Figure 7_New/Figure 7E/WT_CC thickness_P14.tif]

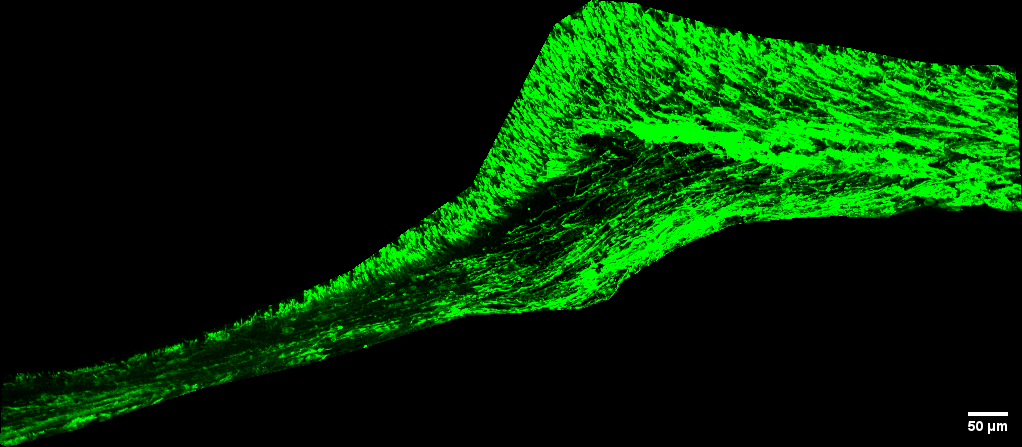

Supplement: Supplementary file 12 — Source data Fig. 7 [file 44321_2025_367_MOESM12_ESM.zip › Figure 7_New/FIgure 7F/NDUFS4 KO_MBP_Mean fluorescence intensity_P14.tif]

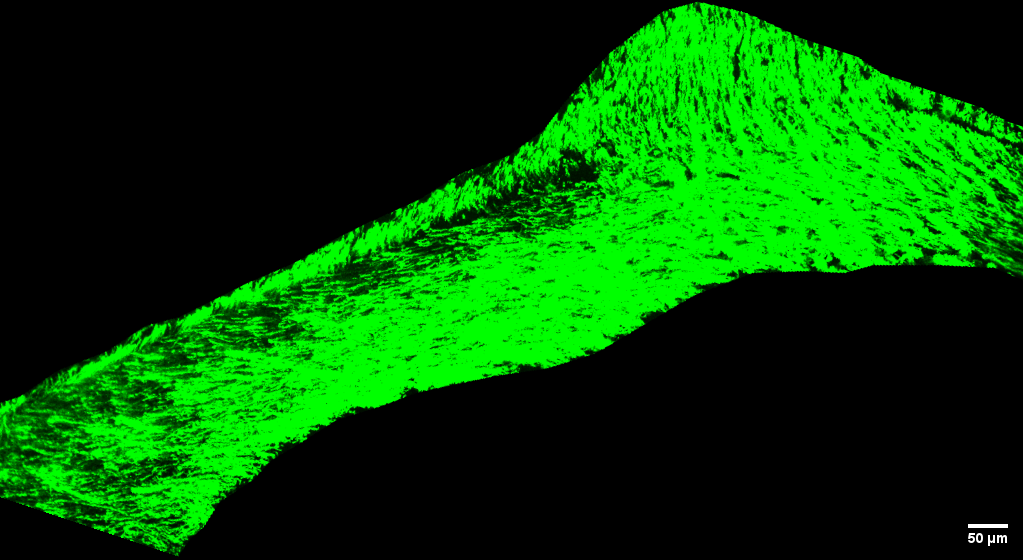

Supplement: Supplementary file 12 — Source data Fig. 7 [file 44321_2025_367_MOESM12_ESM.zip › Figure 7_New/FIgure 7F/WT_MBP_Mean fluorescence intensity_P14.tif]

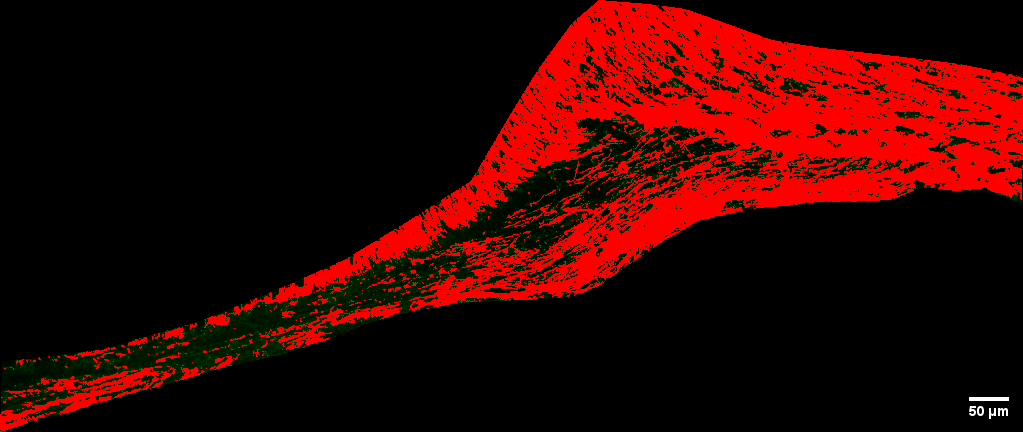

Supplement: Supplementary file 12 — Source data Fig. 7 [file 44321_2025_367_MOESM12_ESM.zip › Figure 7_New/Figure 7H/NDUFS4 KO_MBP_Area coverage_P14.tif]

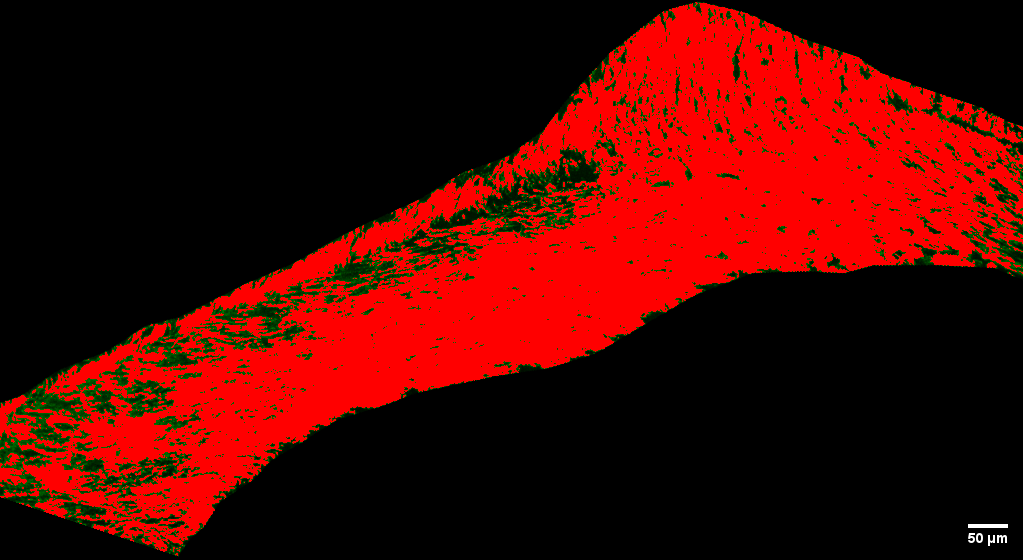

Supplement: Supplementary file 12 — Source data Fig. 7 [file 44321_2025_367_MOESM12_ESM.zip › Figure 7_New/Figure 7H/WT_MBP_Area coverage_P14.tif]

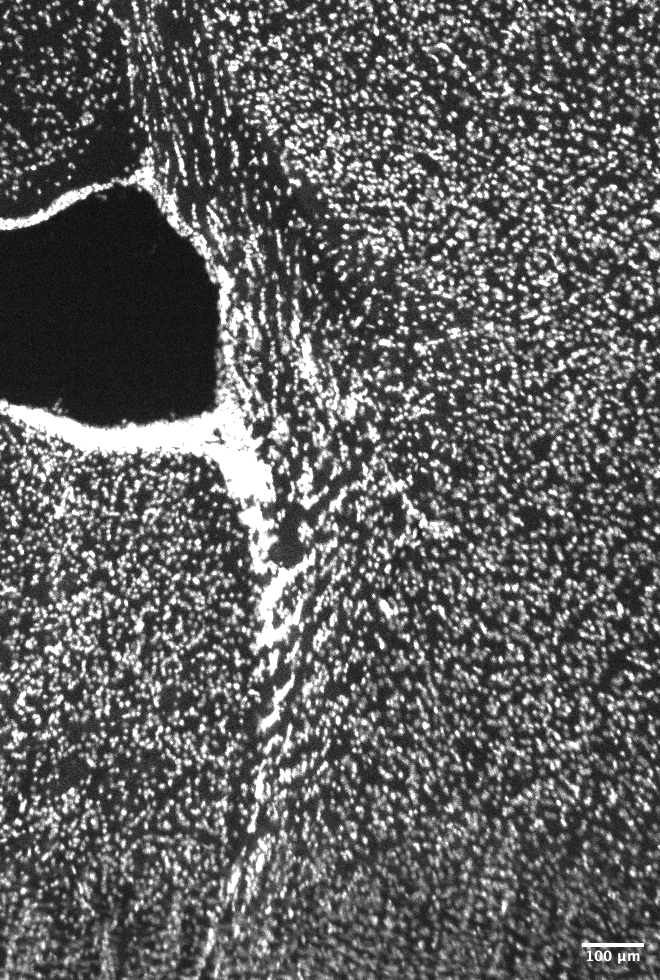

Supplement: Supplementary file 12 — Source data Fig. 7 [file 44321_2025_367_MOESM12_ESM.zip › Figure 7_New/Figure 7L/NDUFS4 KO_CC thickness_P30.tif]

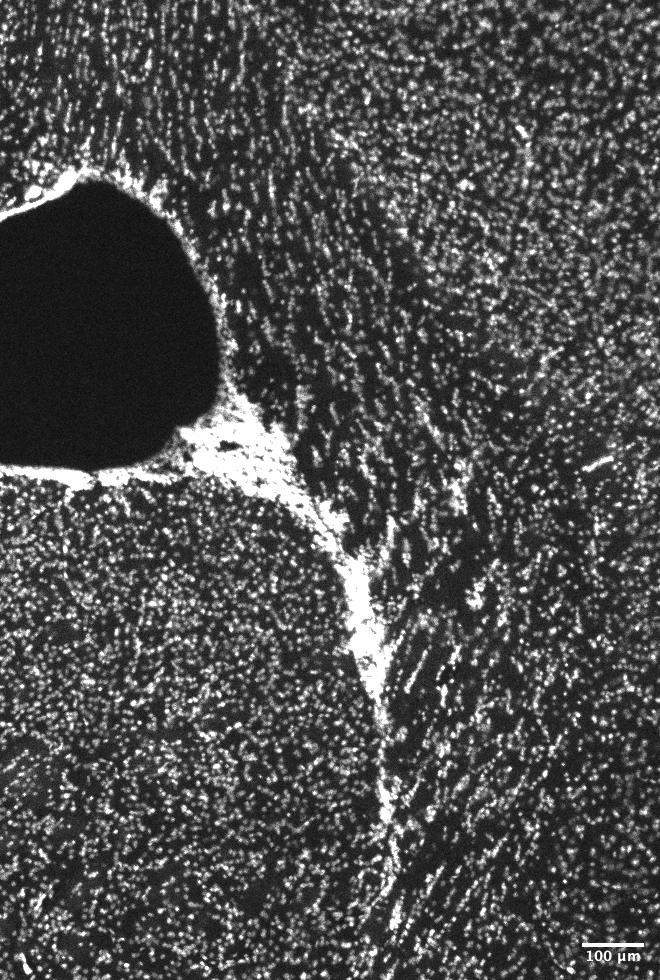

Supplement: Supplementary file 12 — Source data Fig. 7 [file 44321_2025_367_MOESM12_ESM.zip › Figure 7_New/Figure 7L/WT_CC thickness_P30.tif]

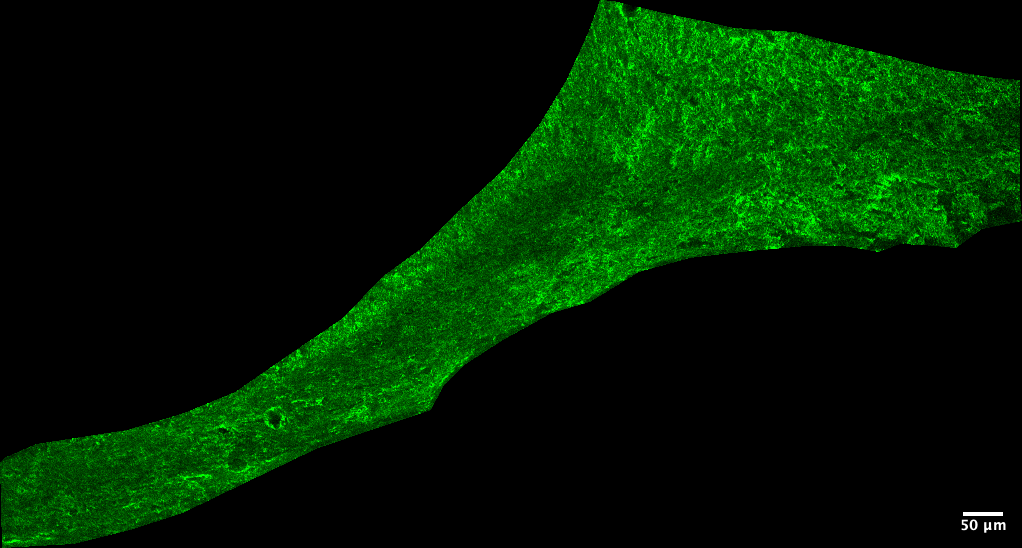

Supplement: Supplementary file 12 — Source data Fig. 7 [file 44321_2025_367_MOESM12_ESM.zip › Figure 7_New/Figure 7M/NDUFS4 KO_MBP_Mean fluorescence intensity_P30.tif]

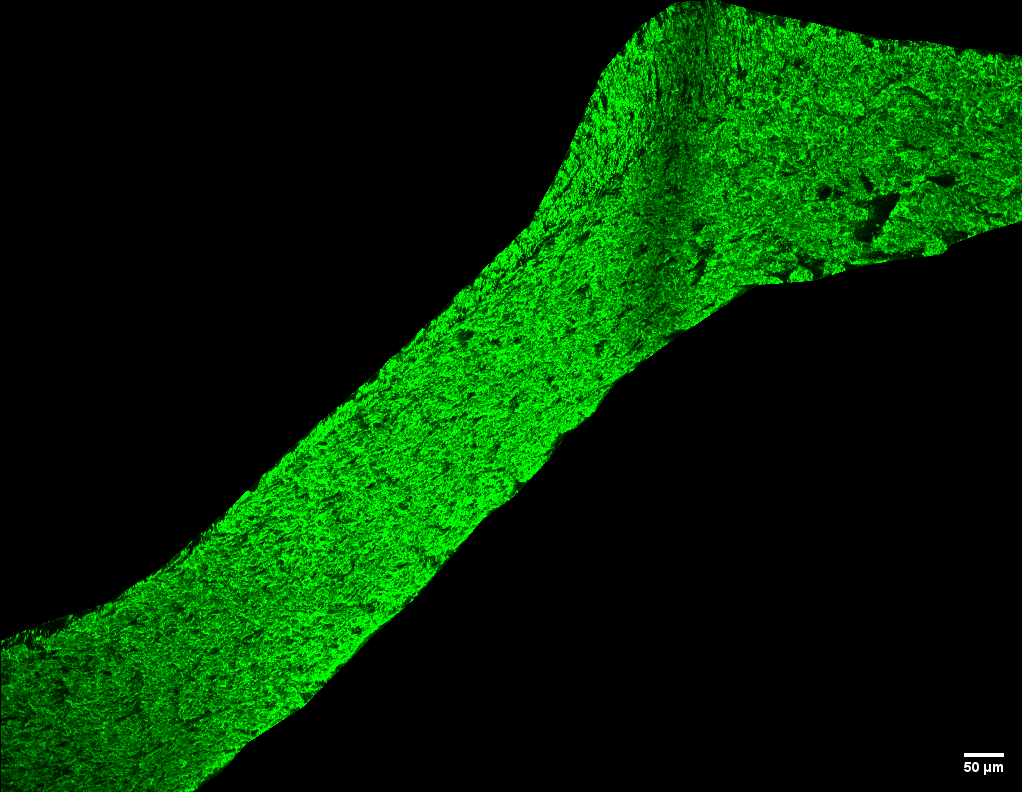

Supplement: Supplementary file 12 — Source data Fig. 7 [file 44321_2025_367_MOESM12_ESM.zip › Figure 7_New/Figure 7M/WT_MBP_Mean fluorescence intensity_P30.tif]

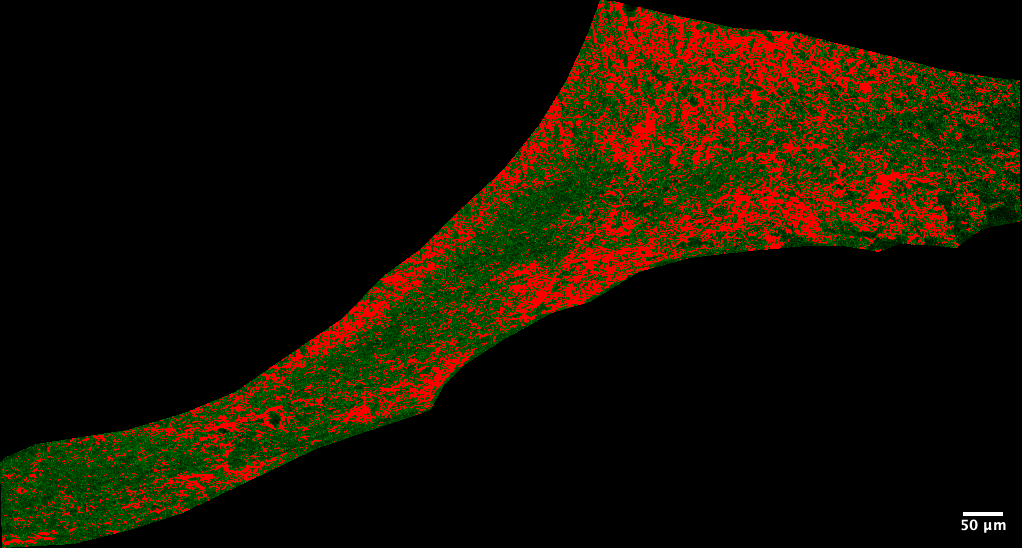

Supplement: Supplementary file 12 — Source data Fig. 7 [file 44321_2025_367_MOESM12_ESM.zip › Figure 7_New/Figure 7O/NDUFS4 KO_MBP_Area coverage_P30.tif]

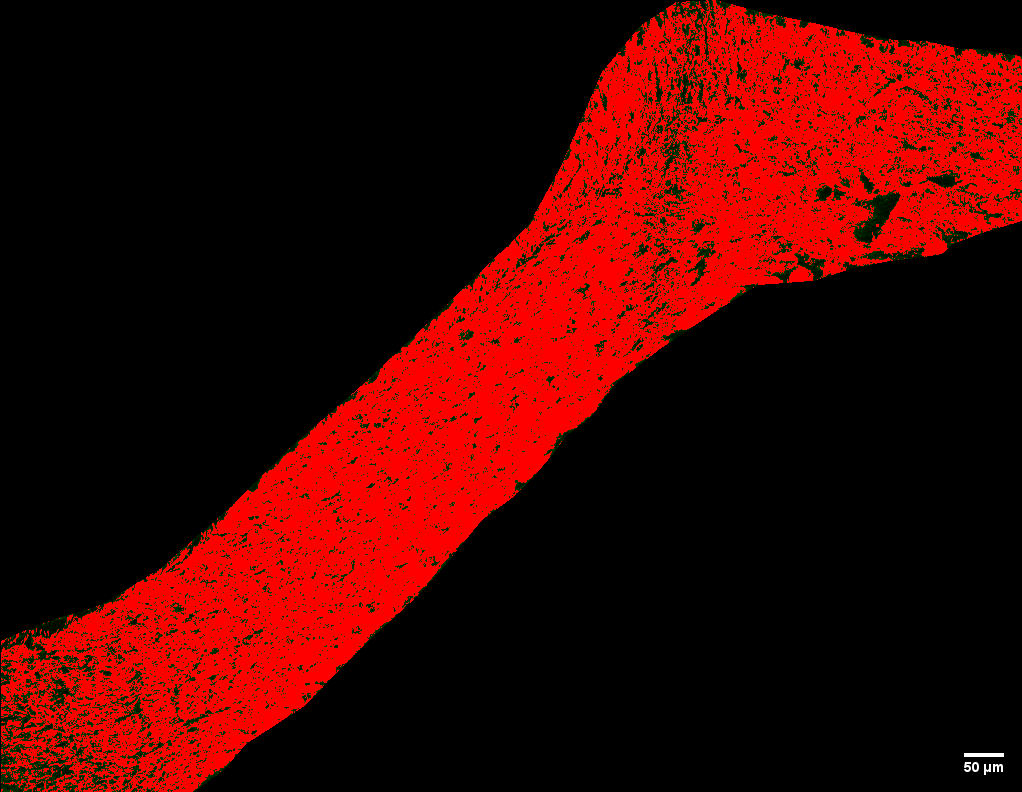

Supplement: Supplementary file 12 — Source data Fig. 7 [file 44321_2025_367_MOESM12_ESM.zip › Figure 7_New/Figure 7O/WT_MBP_Area coverage_P30.tif]
