# Supplementary material for: Impaired Complex I dysregulates neural/glial precursors and corpus callosum development revealing postnatal defects in Leigh syndrome mice
Source: EMBO Mol Med. 2025 Dec 22;18(2):677–701. doi: 10.1038/s44321-025-00367-4 (PMC12905379; doi:10.1038/s44321-025-00367-4)
Supplement: Supplementary file 9 — Source data Fig. 4 [file 44321_2025_367_MOESM9_ESM.zip › Figure 4/Figure 4G/Compositional Analysis summary.docx]

Compositional Analysis summary:

Data: 2 samples, 9 cell types

Reference index: 1

Formula: Condition

Intercepts:

Final Parameter Expected Sample

Cell Type

qNSCs 3.430 725.418824

aNSCs 3.281 624.998454

TAPs 2.599 316.002200

m TAPs 2.994 469.068670

early NBs 4.068 1372.994193

late NBs 1.597 116.018444

Inhibitory Neurons 2.617 321.741741

Excitatory Neurons 0.585 42.171691

Cortical interneurons 1.884 154.585784

Effects:

Final Parameter Expected Sample log2-fold change

Covariate Cell Type

Condition[T.Knockout] qNSCs 0.000000 750.101444 0.048272

aNSCs 0.000000 646.264237 0.048272

TAPs 0.000000 326.754282 0.048272

m TAPs 0.000000 485.028890 0.048272

early NBs -2.781141 87.976296 -3.964067

late NBs 2.391134 1310.733524 3.497948

Inhibitory Neurons 0.000000 332.689112 0.048272

Excitatory Neurons 0.000000 43.606597 0.048272

Cortical interneurons 0.000000 159.845617 0.048272
