# Supplementary material for: Impaired Complex I dysregulates neural/glial precursors and corpus callosum development revealing postnatal defects in Leigh syndrome mice
Source: EMBO Mol Med. 2025 Dec 22;18(2):677–701. doi: 10.1038/s44321-025-00367-4 (PMC12905379; doi:10.1038/s44321-025-00367-4)
Supplement: Supplementary file 13 — Expanded View Figures [file 44321_2025_367_MOESM13_ESM.pdf]

Expanded View Figures

Figure EV1. Neural stem and progenitor cell spatial distribution between genotypes.

(**A**) Graphical depiction of brain regions analyzed in this study generated with Procreate software. The region of interest is colored in blue. (**B–D**) Bregma distribution of $SOX2^+$ cells between WT and NDUFS4 KO mice in the SVZ at (**B**) P14, (**C**) P24, and (**D**) P30 ($n = 5$ mice/group). (**B**) $P = 0.2728$ (1.53), 0.9332 (1.23), 0.0018 (0.93), 0.0002 (0.63), <0.0001 (0.33). (**E**, **F**) Bregma distribution of SOX^+ DCX^+ cells between WT and NDUFS4 KO mice in the SVZ at (**E**) P24, (**F**) P30 ($n = 5$ mice/group). (**E**) $P = 0.1531$ (1.53), 0.0047 (1.23), 0.5558 (0.93), 0.7651 (0.63), 0.8901 (0.33). (**F**) $P = 0.9211$ (1.53), 0.0188 (1.23), 0.0519 (0.93), 0.0945 (0.63), 0.1319 (0.33). (**G–I**) Number of DCX^+ cells between WT and NDUFS4 KO mice in the SVZ at (**G**) P14, (**H**) P24, and (**I**) P30 ($n = 5$ mice/group). (**G**) $P = < 0.0001$ (1.53), 0.0011 (1.23), 0.0497 (0.93), 0.4618 (0.63), 0.9992 (0.33). In (**B–I**), data represents mean and standard error of mean. Each dot represents one animal. $^*P < 0.05$, $^{**}P < 0.01$, $^{***}P < 0.001$, $^{****}P < 0.0001$, ns not significant (two-way ANOVA).

 © The Author(s)

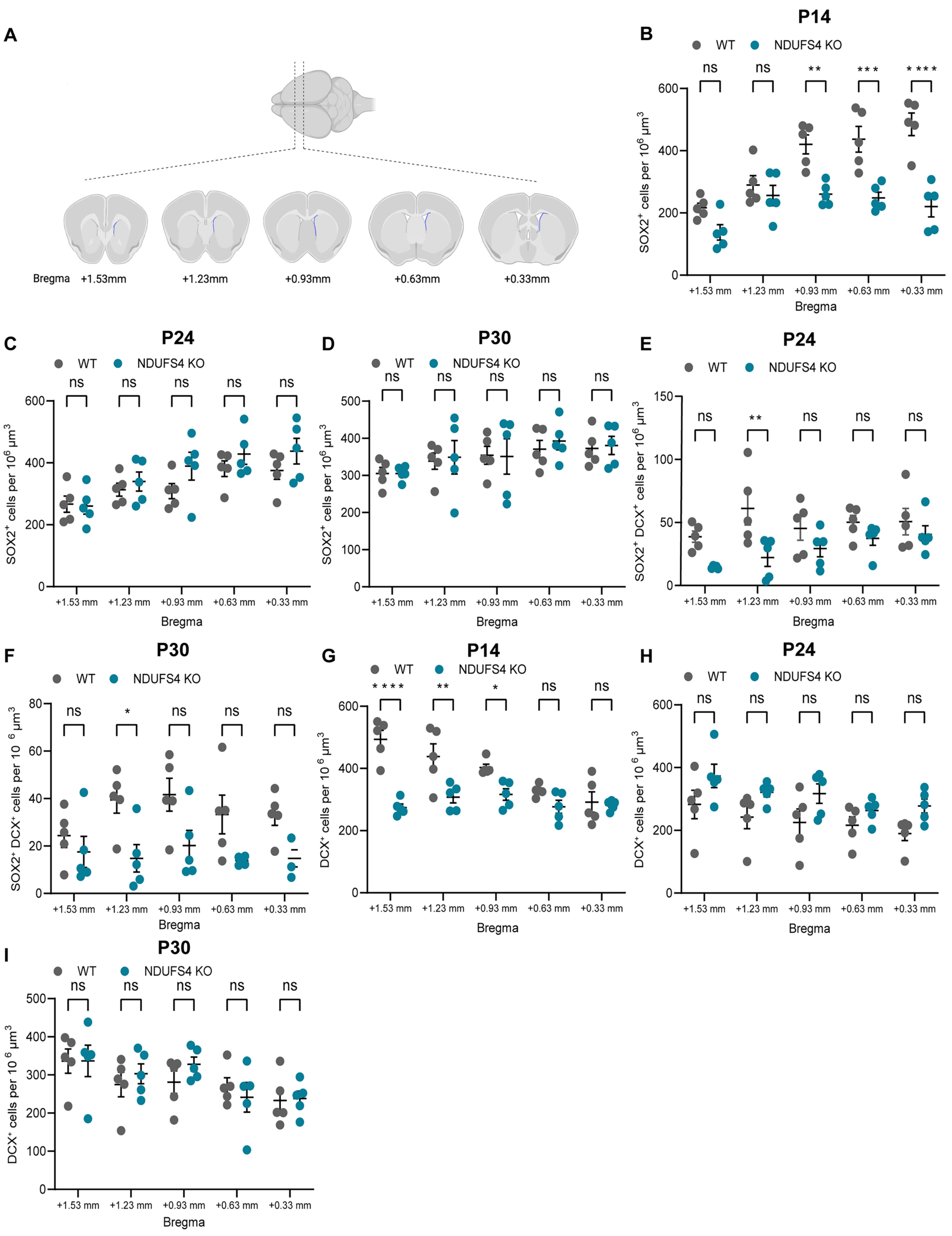
A
Bregma +1.53mm
+1.23mm
+0.93mm
+0.63mm
+0.33mm
B
P14
WT NDUFS4 KO
ns ns ** *** ****
SOX2+ cells per 10^6 µm^3
0 200 400 600
+1.53 mm +1.23 mm +0.93 mm +0.63 mm +0.33 mm
Bregma
C
P24
WT NDUFS4 KO
ns ns ns ns ns
SOX2+ cells per 10^6 µm^3
0 200 400 600
+1.53 mm +1.23 mm +0.93 mm +0.63 mm +0.33 mm
Bregma
D
P30
WT NDUFS4 KO
ns ns ns ns ns
SOX2+ cells per 10^6 µm^3
0 100 200 300 400 500
+1.53 mm +1.23 mm +0.93 mm +0.63 mm +0.33 mm
Bregma
E
P24
WT NDUFS4 KO
ns ** ns ns ns
SOX2+ DCX+ cells per 10^6 µm^3
0 50 100 150
+1.53 mm +1.23 mm +0.93 mm +0.63 mm +0.33 mm
Bregma
F
P30
WT NDUFS4 KO
ns * ns ns ns
SOX2+ DCX+ cells per 10^6 µm^3
0 20 40 60 80
+1.53 mm +1.23 mm +0.93 mm +0.63 mm +0.33 mm
Bregma
G
P14
WT NDUFS4 KO
**** ** * ns ns
DCX+ cells per 10^6 µm^3
0 200 400 600
+1.53 mm +1.23 mm +0.93 mm +0.63 mm +0.33 mm
Bregma
H
P24
WT NDUFS4 KO
ns ns ns ns ns
DCX+ cells per 10^6 µm^3
0 200 400 600
+1.53 mm +1.23 mm +0.93 mm +0.63 mm +0.33 mm
Bregma
I
P30
WT NDUFS4 KO
ns ns ns ns ns
DCX+ cells per 10^6 µm^3
0 100 200 300 400 500
+1.53 mm +1.23 mm +0.93 mm +0.63 mm +0.33 mm
Bregma

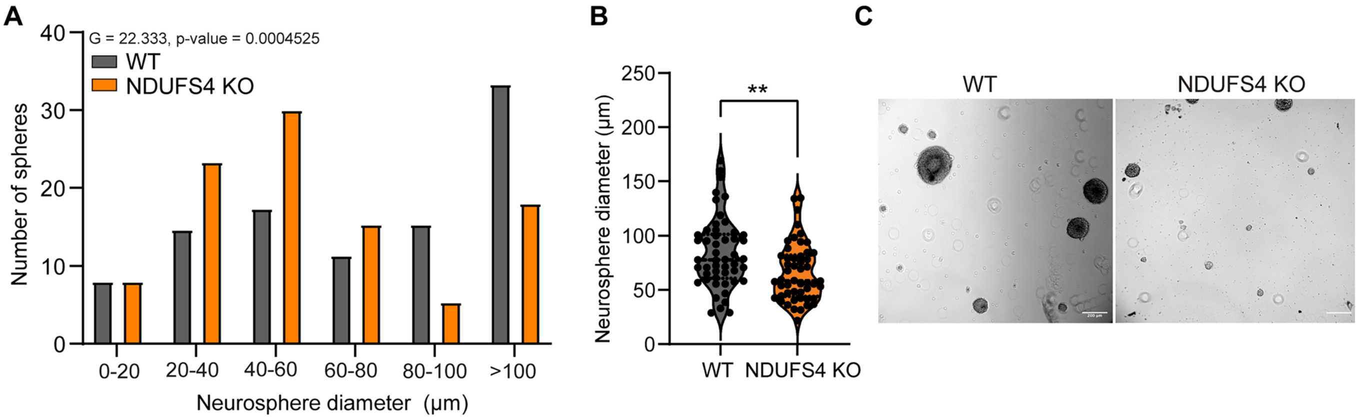

Figure EV2. NDUFS4 KO neural stem cells display a reduced ability to proliferate in vitro.

(**A**) Distribution of neurosphere diameter between WT and NDUFS4 KO animals. Neural stem cells were collected from 3 cultures derived from one animal/genotype. 50 neurospheres were measured per culture. Likelihood ratio test (LRT) was performed to compare the distribution. (**B**) Average neurosphere diameter between WT and NDUFS4 KO. $n = 150$ cells/group. $P = 0.0012$. Each dot represents one neurosphere. $^{**}P < 0.01$ (Student's t test). (**C**) Representative bright-field images for neurospheres. Scale bar = 200 µm.

 © The Author(s)

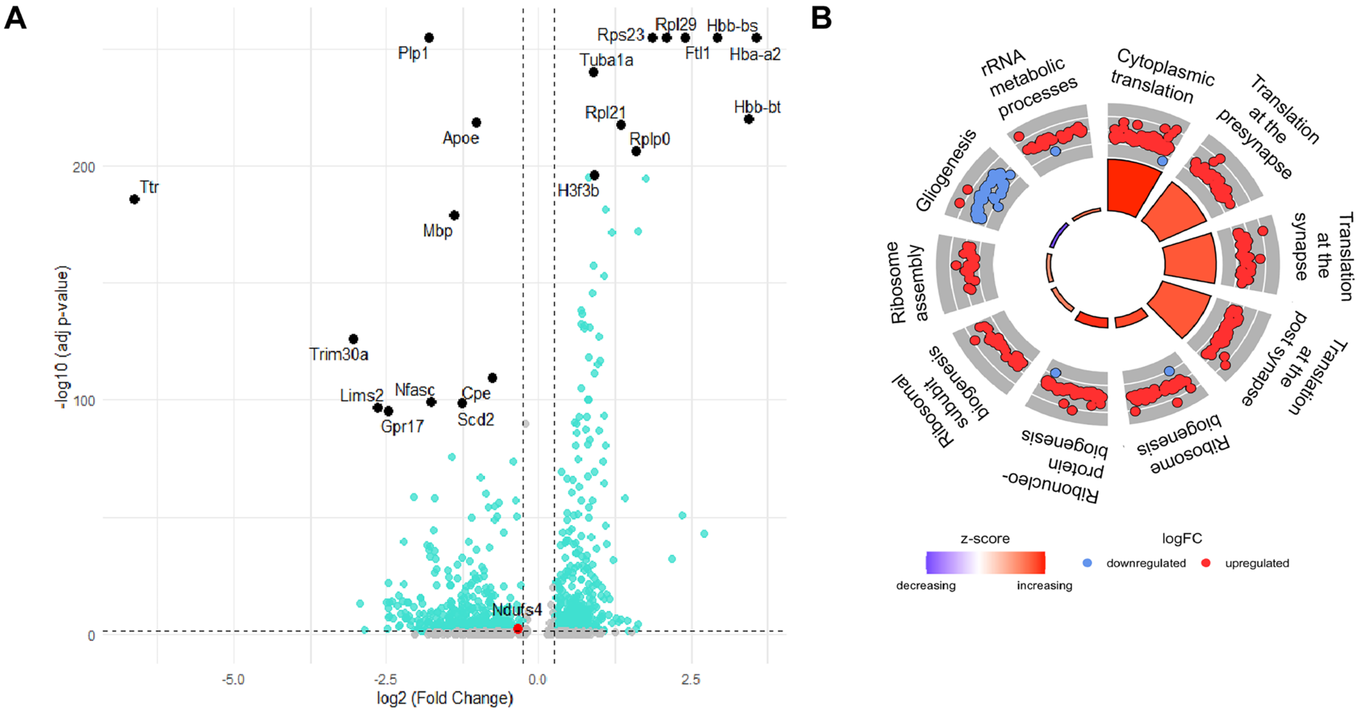

Figure EV3. Upregulated protein translation pathways in NDUFS4 KO neuroblasts.

(**A**) Volcano plot showing fold change of differentially expressed genes in NDUFS4 KO neuroblasts. ($n = 1$ per group, then matched against the other replicate. Ones that are in the same direction were selected). Top 10 up and downregulated mRNAs are labeled (Black circles) and NDUFS4 is highlighted in the red circle. The threshold was set at log2(Fold Change) ≥ +0.25 and ≤ -0.25, and –log10 (adj *P* value) < 1.2991. (**B**) GO circle plot showing top dysregulated biological processes and regulation of associated genes in NDUFS4 KO neuroblasts. In (**A**), Wilcoxon rank-sum test (nonparametric) was performed.

© The Author(s)

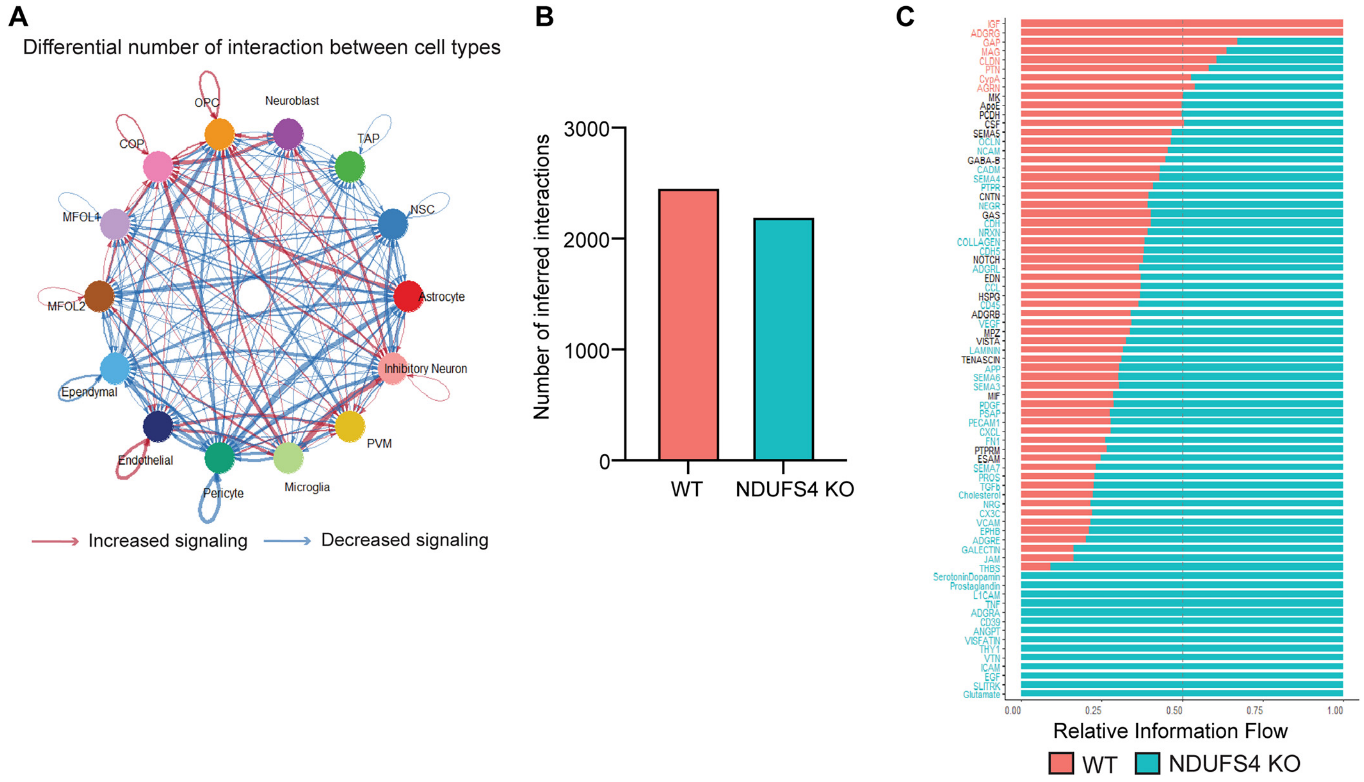

Figure EV4. The number of signaling interactions is decreased in most cell types including neural progenitors in NDUFS4 KO as compared to WT.

(**A**) Differential number of interactions between cell types from NDUFS4 KO. Blue and red lines indicate reduced and increased interactions between cells, respectively. (**B**) Total number of signaling interactions in all cells. (**C**) The significant signaling pathways were ranked based on their differences of overall information flow within the inferred networks between WT and NDUFS4 KO. The overall information flow of a signaling network is calculated by summarizing all the communication probabilities in that network. The top signaling pathways colored by red are more enriched in WT, and the bottom ones colored by cyan were more enriched in the NDUFS4 KO.

 © The Author(s)

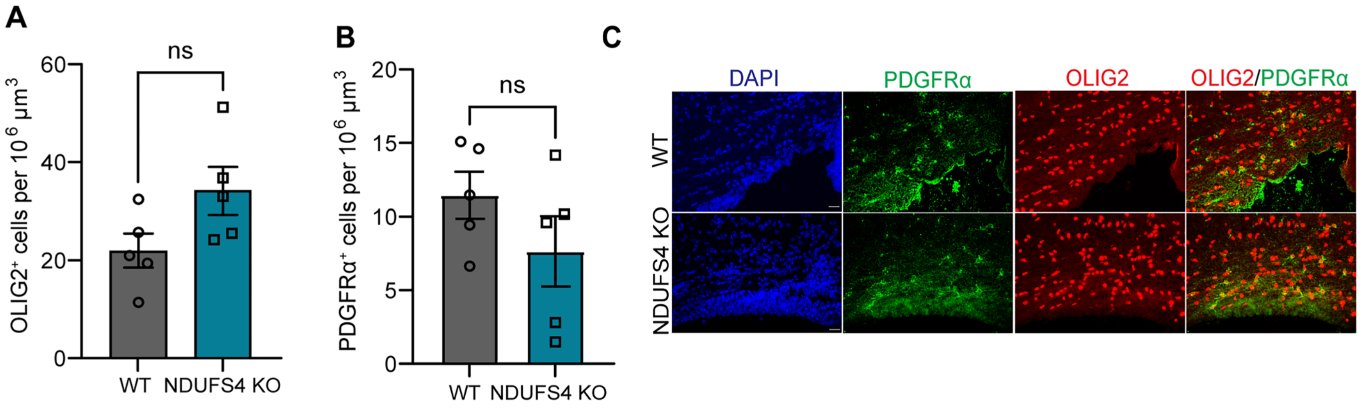

Figure EV5. Oligodendrocytes are not changed in NDUFS4 KO SVZ at early postnatal day P14.

(**A**, **B**) Density of (**A**) oligodendrocytes (OLIG2$^+$) and (**B**) oligodendrocyte progenitors (PDGFRα$^+$) at P14 ($n = 5$ mice/group). (**C**) Representative confocal images of immunohistochemical detection of PDGFRα (green), OLIG2 (red), and DAPI (blue). Scale bar = 20 µm. In (**A**, **B**), bar plot represents mean and standard error of mean. Each dot represents one animal. ns not significant (unpaired *t* test).

© The Author(s)
